# Computational analysis of mRNA expression profiling in the inner ear reveals candidate transcription factors associated with proliferation, differentiation, and deafness

**DOI:** 10.1186/s40246-018-0161-7

**Published:** 2018-06-22

**Authors:** Kobi Perl, Ron Shamir, Karen B. Avraham

**Affiliations:** 1 Blavatnik School of Computer Science, Tel Aviv University, 6997801 Tel Aviv, Israel; 2 Department of Human Molecular Genetics and Biochemistry, Sackler Faculty of Medicine and Sagol School of Neuroscience, Tel Aviv University, 6997801 Tel Aviv, Israel

**Keywords:** Inner ear, Cochlea, Hearing, Balance, Deafness, Transcriptome, Regeneration

## Abstract

**Background:**

Hearing loss is a major cause of disability worldwide, impairing communication, health, and quality of life. Emerging methods of gene therapy aim to address this morbidity, which can be employed to fix a genetic problem causing hair cell dysfunction and to promote the proliferation of supporting cells in the cochlea and their transdifferentiation into hair cells. In order to extend the applicability of gene therapy, the scientific community is focusing on discovery of additional deafness genes, identifying new genetic variants associated with hearing loss, and revealing new factors that can be manipulated in a coordinated manner to improve hair cell regeneration. Here, we addressed these challenges via genome-wide measurement and computational analysis of transcriptional profiles of mouse cochlea and vestibule sensory epithelium at embryonic day (E)16.5 and postnatal day (P)0. These time points correspond to developmental stages before and during the acquisition of mechanosensitivity, a major turning point in the ability to hear.

**Results:**

We hypothesized that tissue-specific transcription factors are primarily involved in differentiation, while those associated with development are more concerned with proliferation. Therefore, we searched for enrichment of transcription factor binding motifs in genes differentially expressed between the tissues and between developmental ages of mouse sensory epithelium. By comparison with transcription factors known to alter their expression during avian hair cell regeneration, we identified 37 candidates likely to be important for regeneration. Furthermore, according to our estimates, only half of the deafness genes in human have been discovered. To help remedy the situation, we developed a machine learning classifier that utilizes the expression patterns of genes to predict how likely they are to be undiscovered deafness genes.

**Conclusions:**

We used a novel approach to highlight novel additional factors that can serve as points of intervention for enhancing hair cell regeneration. Given the similarities between mouse and human deafness, our predictions may be of value in prioritizing future research on novel human deafness genes.

**Electronic supplementary material:**

The online version of this article (10.1186/s40246-018-0161-7) contains supplementary material, which is available to authorized users.

## Background

Hearing and balance are fundamental processes that are essential for communication and for orientation within space. The inner ear is composed of the auditory system, which is responsible for hearing, and the vestibular system, which is responsible, in part, for balance. While these systems display extensive similarities, there are also structural and functional differences. The organ of Corti in the cochlea is unique to the auditory system and contains the sensory epithelium responsible for hearing. In contrast, the vestibular system contains five organs: the three semicircular canals lined with cristae sensory epithelium that detect angular acceleration by fluid motion, and the saccule and utricle, which contain the macula sensory epithelium that can sense linear acceleration due to gravity. The development of the inner ear requires a complex dynamic process to produce the final sensory organ with both hearing and balance capabilities [1].

The mouse has long served as a model for studying human inner ear structure and function, in part because of the ability to breed and select offspring with desired traits, including those affecting hearing and balance [2]. More recently, the similarities between the genomes, and the ability to manipulate the mouse phenotype by gene-targeted mutagenesis and genome editing, have reaffirmed the mouse as an ideal vehicle for studying human auditory and vestibular dysfunction [3, 4]. As a result, mouse inner ear development has been studied in detail on a molecular level [5, 6]. This includes the elucidation of transcriptional pathways that govern the differentiation of the otocyst towards sensory or nonsensory regions during early development (reviewed in [7]). A number of temporal and spatial triggers of development and maturation have been characterized, including the molecular controls on patterning, hair bundle height, and numbers of stereocilia. Information about the active transcriptional pathways has laid the groundwork for establishing the nature of the early and late developmental pathways of the inner ear. Mutations in some of these critical developmental genes are now known to lead to defects in the mouse [8] and human inner ear and to cause deafness [9].

Regeneration after cellular damage shares some similarities with normal organ development. In birds, regeneration of hair cells involves proliferation of nearby epithelial supporting cells, which then differentiate to form replacement hair cells and supporting cells [10, 11]. However, while mature mammalian vestibular organs are also able to regenerate at least a subpopulation of hair cells after damage [12–14], the adult cochlea is incapable of any regeneration. It should be noted that there is some evidence that the cochlea may contain supporting cells with the ability to form new hair cells in very young animals [15] or upon misexpression of *Atoh1* [16]. Given the limitations in the mammalian systems, the resemblance of the auditory sensory epithelia and cochlea between birds and mammals [5], and the ability of birds to regenerate hair cells in the cochlea and vestibule, it is relevant to compare the gene expression profiles of the mammalian and avian inner ears. To this end, we applied systemic transcriptomic approaches to decipher the regulatory pathways of the auditory system and make relevant comparisons to the avian transcriptome.

Sensorineural hearing loss most commonly results from degeneration of cochlear hair cells. As mentioned, if these are lost through damage or the natural aging process, they are not replaced. Gene therapy could potentially be used to induce hair cell regeneration [17]. For many tissues, reprogramming and regeneration is achieved by coordinated manipulation of multiple factors. Initial evidence shows this approach might be successful in the cochlea. In embryonic and neonatal mouse cochlear tissue, ectopic expression of *ETV4*, *TCF3*, *GATA3*, *MYCN*, or *ETS2* in combination with *ATOH1* yielded more hair cell-like cells than did overexpression of *ATOH1* alone [18, 19]. The efficacy of these interventions is partial, rendering the search for other transcription factors (TFs) that can be manipulated to enhance this process extremely relevant. As the number of TFs in human is estimated to be in the range of a few thousands [20], one cannot perform an exhaustive experimental search on all possible manipulations of TFs and their combinations. Instead one should focus its efforts on TFs that are more likely to participate in tissue differentiation. In the aforementioned studies [18, 19], the manipulation was performed on TFs that have conserved binding sites near ATOH1 on the POU4F3 gene. Here, we suggest yet another method to identify these candidate TFs, which focus on the concordance between TFs involved in tissue identity in early stages of development, and those participating in avian hair cell regeneration.

The main purpose of this research was to elucidate transcriptional pathways that govern auditory versus vestibular functions or control cell cycle exit. We report the characterization of transcriptional profiles for mouse cochlea and vestibule sensory epithelium at embryonic day (E)16.5 and postnatal day (P)0, time points chosen because they correspond to developmental stages before and during the acquisition of mechanosensitivity [21]. Genes differentially expressed between the tissues, and between the developmental ages, could be associated with the activity of specific TFs. Our analysis identified a number of regulators that are already known, while others are novel. The identified regulators were compared with TFs already known to alter their expression during avian hair cell regeneration [22, 23], allowing us to detect TFs involved either in proliferation or in differentiation of the inner ear. To our knowledge, this is the first study in the inner ear to integrate expression data from a developmental study in the mouse with data from a regeneration experiment in the chick in the search for TFs governing regeneration.

In addition, our analysis identified a number of candidate genes as involved in inner ear defects. For this purpose, we developed a machine learning classifier, which utilized the expression patterns of genes to predict their probabilities of being yet undiscovered deafness genes. Our predictions allow for prioritizing of candidate genes by their probability to be involved in deafness. The development of a classifier for deafness genes is another unique contribution of this study. Our predictions of novel deafness genes and of TFs with a role in regeneration can be helpful in advancing gene therapy research.

## Results

### Tissue source and age are associated with differences in transcription

**Fig. 1:**
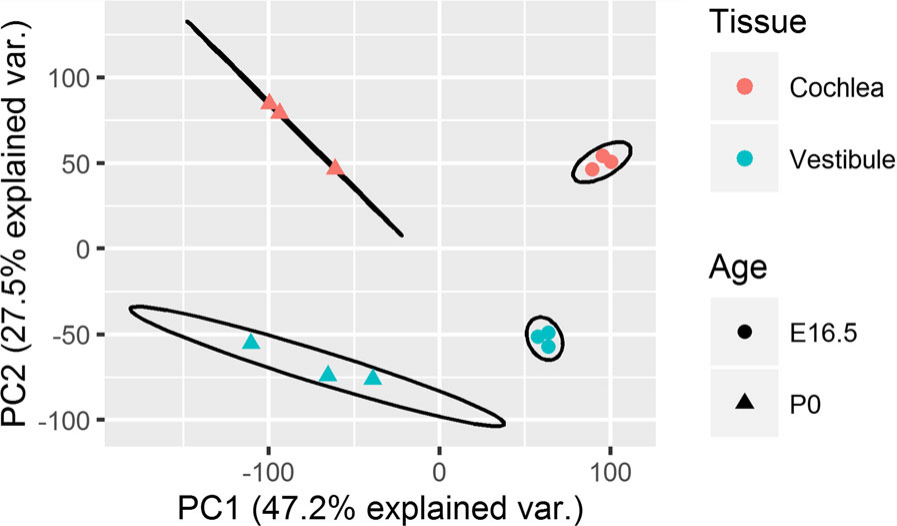
PCA plot comparing samples in the different ages and tissues according to their mRNA expression. The *x*- and *y*-axes are the first and second coordinates, respectively. The samples are colored according to their originating tissue, while the marker shape relates to age. A normal contour line is drawn at 68% probability for each group

Sensory epithelia were dissected from the cochlea and vestibule of mice at two stages of development, embryonic day 16.5 (E16.5) and postnatal day 0 (P0). This was followed by RNA-seq, as previously described [24, 25]. Our analysis identified 39,178 Ensembl genes (including non-coding genes and pseudogenes), 15,206 of which have at least one read per million in three or more of the samples. A principal component analysis (PCA) plot demonstrated four well separated groups (Fig. 1). The first principal component (PC1) explained almost half the variance and is associated with the age of the sample, whereas PC2 explained about a quarter of the variance and is associated with the originating tissue (*F* test on associations, *p* values = 1.99 × 10^−5^, 1.31 × 10^−5^, respectively). Additional PCs were not associated with either tissue or age (*p* value ≥ 0.05). The E16.5 genes displayed a lower intra-group variability than at P0. This might reflect differences in the rate of development of the different organs between mice from the same population in the period between E16.5 and P0.

We used linear mixed models to estimate the percentage of variance that can be attributed to age, tissue, or the interaction of age and tissue (Additional file 1: Supplementary Methods). According to our estimates, the majority of variance can be attributed to either age (44.0 ± 6.5) or tissue (39.6 ± 5.4) (mean percentage ± standard deviation). The remaining non-negligible percentage can be attributed to the interaction term (8.0 ± 1.5), and a model with this interaction term describes the data better according to a restricted likelihood ratio test (*p* value ≤ 2.2 × 10^−16^). Less than 10% of the variance was left unexplained (8.4 ± 1.08).

We selected genes that were differentially expressed between tissues or between ages, and genes for which the interaction of tissue and age was significant in determining expression. Our results identified 3306 upregulated genes and 6890 downregulated genes at P0 compared to E16.5. Four thousand one hundred fifty-nine genes were found to be upregulated and 2382 were downregulated in the vestibule compared to the cochlea. For 745 genes, the cochlea to vestibule expression ratio increased over development, and it decreased for 1211 genes. We performed gene ontology (GO) and Kyoto Encyclopedia of Genes and Genomes (KEGG) enrichment analyses on genes from the six identified sets (Additional file 2: Table S1). The enrichment results are summarized below.

### Expression changes with age

Genes that were upregulated at E16.5 are enriched for terms related to cell cycle, DNA replication, cytoskeleton organization, and other terms that are in accordance with a highly proliferative state. In contrast, genes that were upregulated at P0 are enriched for ribosomes, indicating high protein synthesis, mainly of plasma membrane and extracellular matrix proteins. The lipid and oxphos-related metabolic activities are also high in this group. The cells at this stage of development are more adhesive, communicate more with one another, and are more responsive to external cues. They are also responsive to a variety of signaling receptors, including calcium signaling, and have high ion transport activity. The upregulated terms are typical of a less proliferative environment, where the highly expressed genes promote homeostatic processes and inhibit peptidase activity. Some terms show signs of cell specialization, in terms of sensory perception, cartilage-related metabolism, and the regulation of ossification; the last might indicate a cross-talk between sensory epithelium cells and endochondral cells. Another marker for the more differentiated state is an up-regulation of the MHC protein complex. In summary, the enrichment suggests that the inner ear is in a more proliferative state at E16.5 than at P0, whereas at P0 the tissues are more differentiated and exhibit specialization for sensory perception.

KEGG enrichment generally confirmed the aforementioned differences and provided more details regarding specific metabolic processes activated at P0. For example, we could attribute the enriched lipid metabolism to sphingolipids, arachidonic acid, and retinol, the enriched aminoglycan metabolism to glycan degradation, and the biosynthesis of chondroitin and keratan sulfate. Pathways enriched at P0 suggest that the activity of the immune system increases during development, with leukocytes migrating into the tissue and intercellular communication using cytokines. As the complement and coagulation cascades and the renin-angiotensin system are also enriched at P0, we can hypothesize that the inner ear is more exposed to blood circulation at this age.

### Expression change between tissues

According to the enrichment analysis (Additional file 2: Table S1), a number of the differentially expressed (DE) genes in both the cochlea and vestibule are involved in signal transduction. In the cochlea, the majority of the signaling is mediated by voltage- and ligand-gated ion channels and can be attributed to neuron-neuron synaptic transmission. In agreement with this finding, other upregulated activities are neurogenesis and neuron projection. In contrast, the signaling in the vestibule is probably required for the coordination of both innate and acquired immune responses, an observation that relates to the main function enriched in this tissue. The signaling, some of which involves purinergic receptors, plays a role in the response to external stimulus and stress, and also in taxis. Another function enriched in the vestibule is locomotion, with the cilium and the axoneme being two enriched cellular components related to the movement of the hair cells’ stereocilia. The vestibule is richer in blood vessel formation and hematopoiesis, and the extracellular matrix is more evolved than in the cochlea. Together with the high immune-related activity, these factors may explain why the vestibular cells are more adhesive. We also detected enrichment for replacement ossification, suggesting the development of bone. As a generalization, upregulated genes were associated with neurological terms in the cochlear, but to vascular, structural, and immunological terms in the vestibule. This partitioning was not perfect as we could detect enrichment for mesenchymal cell differentiation in the cochlea, and 3.1% of the upregulated genes in the vestibule were annotated for a role in sensory perception.

The KEGG enrichment data also agreed with the characterization of the cochlea as more neurological versus a more vascular vestibule. In addition, the data provided more information about the typical signaling in each apparatus. Neuroactive ligand signaling was identified in both, although the cochlea was associated with the TGF-beta, MAPK, and ErbB signaling pathways, while cytokine-mediated, calcium, and Toll-like receptor signaling were more important in the vestibule. Three pathways shown to be unique to the cochlea affect cell proliferation, survival, differentiation, and migration [26–28], suggesting that these developmental processes are more activated in the cochlea. Other unique metabolic pathways enriched in the cochlea were O-glycan and chondroitin sulfate biosynthesis. The vestibule, on the other hand, was enriched for glycan degradation and metabolic pathways concerning arachidonic acid, retinol, and glutathione.

### Tissue expression ratio change with age

Genes for which the cochlea to vestibule expression ratio increased with age $\left(\frac{Cochlea}{Vestibule}\uparrow \right)$ were enriched for processes related to sensory perception and central nervous system development, as well as signaling through G-coupled receptors, ligand-gated ion channels, or calcium. Accordingly, a significant number of genes were annotated to be in the apical part of the cell. Other genes annotated to the extracellular region might mediate the biological adhesion, which increases during development. Another enriched component was identified as the sarcomere, which most closely resembles the stereocilia in the inner ear.

**Fig. 2:**
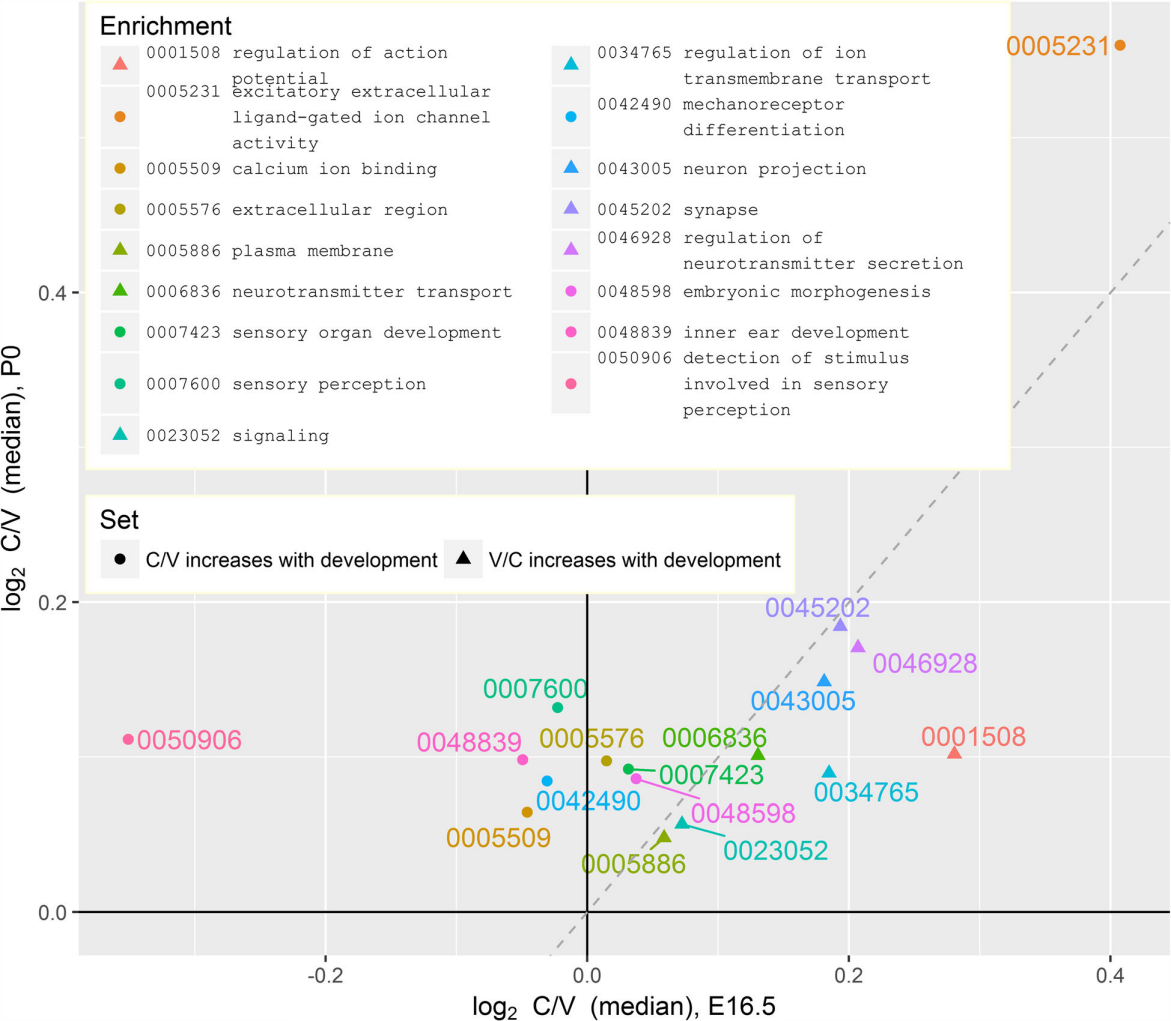
GO terms enriched for genes affected by age-tissue interaction. The median cochlea to vestibule (C/V) expression ratios of genes annotated for GO terms at P0 (*y*-axis) against E16.5 (*x*-axis). Circles mark GO terms enriched for genes with increased C/V ratios between E16.5 and P0, and for which the ratios of all annotated genes are higher at P0 than at E16.5. Triangles mark GO terms with parallel properties for the reciprocal ratio (V/C)

We can envision two possible scenarios for each of these enrichments. The first option is that genes annotated for enrichment are upregulated in the cochlea at E16.5 and the gap between the cochlea and the vestibule increases during development. The second option is that these genes are upregulated in the vestibule at E16.5 and the gap between the cochlea and the vestibule decreases during development. To distinguish between the two, we compared the expression of all genes that are annotated for each GO term. The median expression log-ratio between the cochlea and the vestibule at P0 was plotted against the value of the same parameter at E16.5 (Fig. 2, circles). The plot only contains the terms for which the gap between the cochlea and the vestibule significantly increases with age. More precisely, only terms for which the log-ratios at P0 were larger than their paired values at E16.5 were included (Wilcoxon signed rank test, *q* values ≤ 0.05).

Interestingly, the vestibule is appeared to be more specialized for sensory perception at E16.5 than the cochlea, as manifested by a negative median log-ratio for terms sensory perception, mechanoreceptor differentiation, and detection of stimulus involved in sensory perception. However, by P0, the cochlea surpassed the vestibule in all of these fields. In contrast, ligand-gated ion channel activity was already higher in the cochlea at E16.5, and the gap only increased with development.

Genes for which the vestibule to cochlea ratio increased with age $\left(\frac{Vestibule}{Cochlea}\uparrow \right)$ were enriched for signaling, neuron projection, neurotransmitter transport, and secretion. These are all functions that are higher in the cochlea at E16.5, and for which the difference between the vestibule and the cochlea decreases with time (Fig. 2, triangles).

### Deafness genes can be predicted using expression patterns

A list of 140 genes associated with human deafness was compiled from a public dataset (http://hereditaryhearingloss.org/; Additional file 3: Table S2). Expression data for 130 orthologous mouse genes are available in our dataset. Of these genes, mutations in 25 orthologs are associated with syndromic deafness in human, 96 with non-syndromic deafness, and nine with both types of deafness and are treated as syndromic deafness genes (DGs) in subsequent analyses. It should be noted that we found no ortholog for any of the five mitochondrial DGs.

**Fig. 3:**
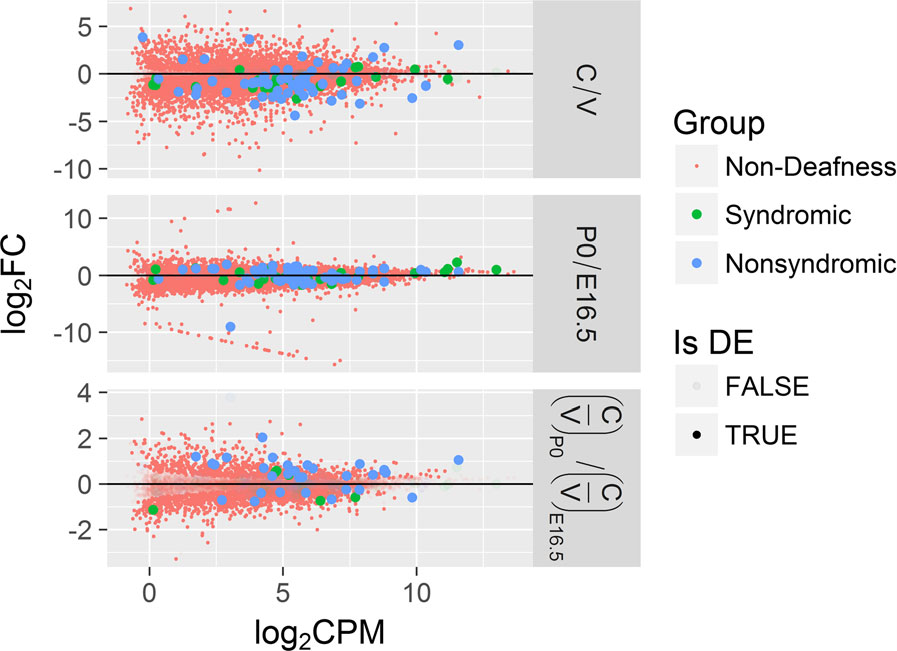
FCs against average expression for deafness and non-deafness genes. Each point represents a gene. The logarithm of the FCs of genes between tissues (upper), ages (middle), and age-tissue combinations (lower) are plotted against their averaged expression across samples (in log counts per million [CPM]). Transparent points correspond to genes that are not differentially expressed in the comparison. Deafness genes are marked with larger points and are colored based on the type of deafness involved

We observed general patterns of expression for these syndromic and non-syndromic DGs. First, when comparing vestibular and cochlear expression, the absolute values of the fold change (FC) of the DGs were higher than for the background FCs (*p* value = 1.98 × 10^−5^, one-sided Wilcoxon rank sum test; Fig. 3, upper subfigure). In addition, the absolute FCs of non-syndromic DGs were slightly higher than the FCs of syndromic DGs (*p* value = 7.00 × 10^−2^, same test). That is, DGs tend to be tissue-specific, with the non-syndromic genes possibly being even more specific. Interestingly, the majority of the DE DGs were higher in the vestibule than in the cochlea, despite the acknowledged role of the cochlea in hearing (57 out of 76, *p* value = 2.19 × 10^−5^, two-sided proportion test).

Second, when comparing P0 and E16.5 expression, DGs tended to have higher FCs compared to background FCs (*p* values = 6.32 × 10^−6^, one-sided Wilcoxon rank sum test; Fig. 3, middle subfigure). This indicates that their expression tends to increase with development. Third, their cochlea to vestibule expression ratio tended to increase with development compared to background (*p* values = 5.15 × 10^−6^, same test; Fig. 3, lower subfigure). Moreover, the increase in the ratio of non-syndromic DGs was higher than that for syndromic genes (*p* value = 3.48 × 10^−3^, same test).

### Deafness gene prediction

We used the three types of FC and the averaged expression (see the “Methods” section), to build a classifier that can predict whether a gene is a DG. The classifier achieved a ROC score of 0.66 ± 0.04 across repeated training/test splits. A ROC score of greater than 0.5 indicates that the expression data have some predictive value for the relation of a gene to deafness. This classifier performs better than a similar version that used the averaged RPKM values in each condition (ROC score 0.60 ± 0.05). Removing one or more of the four feature types from the original classifier resulted in a lower score.

It must be appreciated that genes not marked as DGs might still represent undiscovered DGs. For this reason, it was important to train our classifier to distinguish between known DGs and genes with an unknown role in deafness. The first group of genes is termed positive, and those in the second group are classified as unlabeled. We wished to adapt our positive unlabeled (PU) classifier to output the probability that an unlabeled gene is a positive gene. This type of classification is referred to as transductive PU learning [29]. Supposing that the known DGs are a random subset of all DGs, i.e., the features we explore impose no bias over which of the positive genes are labeled, then, the probability that the PU classifier assigns to the positivity of new genes both (i) correctly ranks the genes and (ii) the probabilities are only off by a constant factor (see [30] for details). We used a bagging-like algorithm similar to the one presented in [29] in order to calculate the probabilities for the set of unlabeled genes. Some modifications in our system are described in the “Methods” section. One main difference between our approach and the previously reported version in [29] was that we kept the same proportion of positive (labeled) samples in the training set as in the test set, whereas in [29], all positive samples were included in training. This property allowed us to address the issue of biases in the probabilities, albeit at the price of losing some predictive power. One source of bias was due to undersampling in the learning process [31]. A second source of bias was the one described above for a PU classifier. We addressed the latter using methods presented in [30].

To gain some insight about the accuracy of our estimator, in spite of the lack of a definitive classification of the unlabeled set, we downloaded lists of genes associated with hearing loss according to the text mining tools DigSeE [32], DisGeNET [33], and DISEASES [34]. We refer to these genes as deafness-associated genes (DAGs). By these means, we obtained 1313 genes that were associated with deafness according to at least one tool. These included 115 known DGs, accounting for 82% of all reported DGs. The respective numbers of mouse orthologs were 1021, 106, and 82%. See Additional file 1: Supplementary Results, Figure S1 for a comparison of the lists of genes provided by the tools.

Applying our bagging-like algorithm resulted in a PU classifier with a ROC score of 0.694 where the probabilities from this native classifier were probably biased upward due to undersampling. Correcting for this bias resulted in a better calibration of the probabilities, as demonstrated by a calibration plot (Additional file 1: Figure S2, left), and the lowering of the Brier score (BS) from 2.07 × 10^−1^ to 8.47 × 10^−3^. We employed three different methods (e1, e2, e3; see [30]) to correct the bias in the probabilities caused by the PU scenario. In order to perform the calibration, all three methods first estimate the probability that a known DG is labeled *p*(*s* = 1| *y* = 1). The estimates for this probability, according to e1, e2, and e3, were 0.032 ± 0.014, 0.022 ± 0.007, and 0.518 ± 0.248, respectively. The estimates made by e1 and e2 support the existence of a few thousand DGs, compared to the few hundred predicted according to e3 (4.1 × 10^3^, 5.9 × 10^3^, and 2.5 × 10^2^, respectively). We believe that given the status of deafness research, the last estimate is the most reasonable. To investigate the issue further, we re-evaluated the calibration of the probabilities produced by each method. For this purpose, we assumed that all the DAGs are in fact deafness genes. With this assumption, the e3 method resulted in the best calibration, as demonstrated by a calibration plot (Additional file 1: Figure S2, right), and the lowest BS (scores 6.64 × 10^−2^, 1.20 × 10^−1^, 2.84 × 10^−1^, 6.45 × 10^−2^ for no fix, e1, e2, and e3, respectively). Hence, we decided to use e3 probabilities in all subsequent analyses and let *p*_*g*_ be the probability that gene *g* is positive according to e3*.*

We then reran our bagging-like algorithm, but this time, we chose to treat a gene *g* as positive with probability *p*_*g*_, and as negative with probability 1 − *p*_*g*_. This reassignment was performed before each iteration. Finally, we recalculated the ROC score of our classifier. In this case, we ignored known DGs in order to make a proper separation between training and test stages. The rerun achieved a slightly better ROC score (0.602 vs 0.600, *p* < 0.05, DeLong’s test for two correlated ROC curves [35]). We chose to continue with the rerun classifier and added a correction for undersampling to the resultant probabilities. The predictions for both human genes and mouse orthologs are available in Additional file 4: Table S3. The 20 mouse genes with the highest predicted probabilities include the known non-syndromic DGs *Smpx*, and *Ptprq*, seven DAGs (*Gfi1*, *Lhx3*, *Erbb4*, *Ephx1*, *Il33*, *Slc52a3*, and *Ttr*), and nine genes not associated with deafness (*Mlf1*, *Nell1*, *Espnl*, *Rbm24*, *Lrrc10b*, *Agr3*, *Tgm2*, *Id4Cd164l2*, and *Faim2*).

**Fig. 4:**
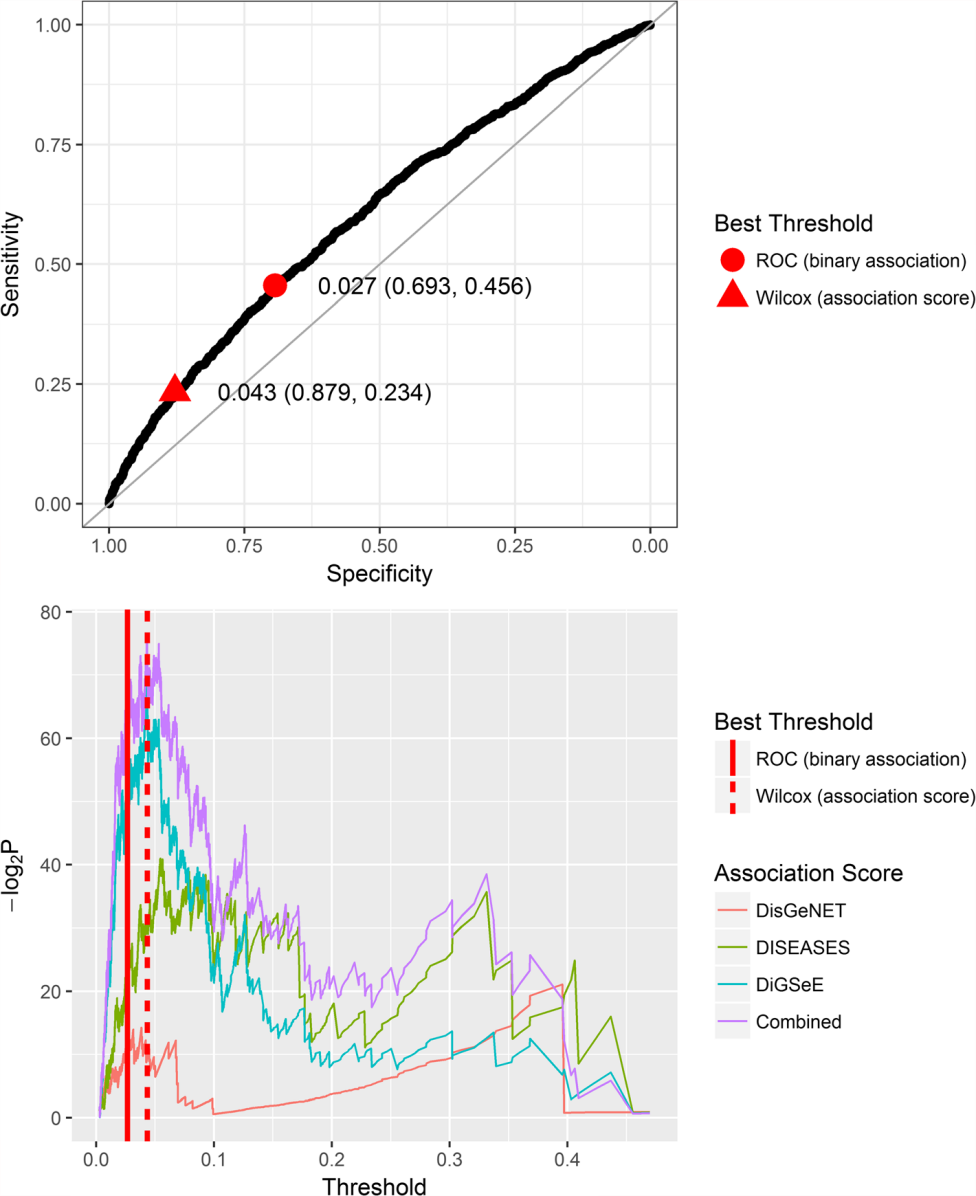
Choosing a threshold probability for discrimination of deafness-associated genes. The two plots demonstrate the effect of choosing a threshold on the balance between sensitivity and specificity of prediction. Top: a standard ROC curve. A gene associated with deafness according to any of the text mining tools is considered a positive gene, all others are considered negative. Bottom: threshold determination based on a comparison of association scores. At each threshold, significance according to the Wilcoxon rank sum test is assigned to the difference between the association scores of genes above the threshold and all other genes. A higher −log_2_*P* value indicates a more significant difference, in the direction of higher association scores for genes above the threshold. Line color indicates the source of the association scores used. Each graph has two thresholds marked. The first is the threshold value for which the sum of specificity and sensitivity of the ROC curve is highest (upper, circle shape; lower, solid vertical line). The second is the threshold value for which the Wilcoxon test is most significant for the “Combined” association score (upper, triangle shape; lower, dotted line)

For the purpose of selecting a discrimination threshold for our binary classifier, we can consider two plots, which demonstrate how well our classifier predicts DAGs (again while ignoring known DGs). The first is a ROC curve, which visualizes the balance between specificity and sensitivity (Fig. 4, top). The threshold maximizing the sum of these two parameters is suggested as a candidate threshold. A disadvantage of a ROC curve, in our context, is that it ignores the association scores provided by the text mining tools. In order to account for these scores, we can consider a range of values of the threshold and use a non-parametric test (one-tailed Wilcoxon rank sum test) to compare the association scores of the genes with probabilities higher than the threshold, with all the others. We hypothesized that genes above the “right” threshold would tend to have higher association scores. We analyzed the association scores from each tool separately and together (see the “Methods” section) and plotted −log_2_*P* − value against the threshold (Fig. 4, bottom). The value giving the lowest *p* value for the combined scoring was proposed as a candidate threshold. Four thousand six hundred seventy-four and 1934 genes passed the thresholds suggested by the ROC curve (0.027) and the Wilcoxon test (0.043), respectively. Other thresholds may also be considered, depending on the required number of candidates, specificity, and sensitivity. We recommend choosing thresholds that give local maxima on either curve (available in Additional file 4: Table S3).

### Transcription factors affecting expression

When we screened for enrichment of transcription factor (TF) binding sites in three sets of DE genes (Additional file 5: Table S4) we could identify six motifs that were associated with changes in expression during development, 43, between tissues, and 10, across an age-tissue interaction (i.e., the change in the cochlea to vestibule expression ratio throughout development). This 7-fold increase in tissue-specific motifs over those associated with a developmental stage was very surprising, in view of the fact that the absolute number of tissue-specific DE genes identified was about 35% less than the number that changed during development. In total, we identified 50 unique motifs across all comparisons and manually connected them to 64 mouse TFs (i.e., a few motifs were associated with multiple TFs).

For each TF, we tested whether the TF gene itself was DE under the same conditions as the gene it regulates (Additional file 5: Table S4). This property interests us for three reasons: (i) It indicates whether the regulation of the TF activity is (at least partially) transcriptional. Knowing how a TF is regulated makes it a better candidate for experimental interventions. (ii) The direction (upregulation or downregulation) in which a TF is DE implies whether it functions as a repressor or an activator. (iii) It strengthens our faith that the associated motif is important for regulation, and not a false-positive. In our analysis, 30 of the 64 TFs identified were DE (in at least one comparison).

In order to investigate how the levels of the TFs affect their targets, we plotted the median FC of *all* targets of a specific motif, against the median FC of the TFs associated with that motif (Additional file 1: Figure S3). In all cases, we observed a positive, although insignificant, correlation between the two values (Pearson’s *r =* 0.51, 0.05, or 0.52 for the comparisons between tissues, across age, and for age-tissue interaction, respectively; combined *p* value (22) = 0.15) [36]. Among the factors that contribute to the incomplete correlation is the post-transcriptional regulation of TFs, which reduces the correlation between the transcript levels and TF activity. In addition, while most TFs activate the transcription of the targets, certain factors can repress the transcription of some or all of their targets. Moreover, taking the median FC of the TFs associated with a motif ignores the possibility of complex interrelationships, such as the ability of a subset of the TFs to activate transcription alone (an example for the motif AHRHIF is discussed later).

The TFs identified as being associated with development were compared with TF genes shown to change their expression during avian regeneration of inner ear sensory epithelia in one of two experiments conducted in chick. The first experiment measured expression of TFs after either laser “wounding” cultured sensory epithelia or treating inner ear organs with the ototoxic antibiotic neomycin [22]. The sampling time points after the laser lesion (30 min, 1 h, 2 h and 3 h) were chosen in order to provide insights into the very early signaling events that occur after epithelial injury, while the sampling time points after a 24-h incubation of inner ear organ cultures with neomycin (immediately after incubation, and after 24 and 48 h in neomycin-free medium) were chosen to cover the period of the S-phase entry by supporting cells, which peaks at about 48 h after ototoxic injury in vitro. Unlike the first experiment, which measured responses to lesions in both cochlear and vestibular cultures, the second regeneration experiment [23] from the same group only measured expression in chick utricles. Also, the second experiment only explored the effect of an ototoxic antibiotic on gene expression. Still, this study had several advantages over [22]. First, it followed the expression changes across a 7-day time course, with more frequent measures in the 48–72 h window of regeneration, a period characterized by highly dynamic patterns of expression for many genes. Second, it employed RNA-seq instead of microarrays for the expression measuring, allowing the authors to obtain a comprehensive transcriptome, instead of specifically focusing on transcription factors expression as was done in the first experiment.

The comparison of the TFs was designed to detect pathways that are common to inner ear development and regeneration and specifically to reveal genes essential for either proliferation of supporting cells, or for transdifferentiation to hair cells. Out of 712 DE TFs in the first regeneration experiment [22], we mapped 596 to orthologous mouse genes. Intersection with our list of 64 TFs yielded 33 TFs that are involved in both development and regeneration (Additional file 1: Figure S4). Significantly, eight of these are also DE. The overlap with the later avian transcriptome experiment [23] was far more limited. Out of 212 DE TFs found in the experiment, we mapped 208 to orthologous mouse genes, of which only six appear also in our list of TFs (Additional file 1: Figure S5), and five of them are also DE. The TFs *SMAD9* and *SPI1* were DE in both avian experiments and were associated with enriched motifs in our developmental study.

Finally, we performed a comprehensive literature search for the motifs found in the context of inner ear development. For a small subset, the results are detailed in the following sections, with a more complete list available in Additional file 1: Supplementary Results.

### Transcription factors affecting expression changes with age

The set of genes that were upregulated at E16.5 was enriched for binding sites for the motifs: Elk-1, Nrf-1, E2F-1, E2F, NF-Y, and AHRHIF of which, the subset Elk-1, Nrf-1, NF-Y, E2F-1 and some TFs associated with the motifs AHRHIF (*Arnt* and *AhR*) were upregulated together with their regulated genes. Upregulation of *Hif1a*, another motif associated with AHRHIF, could be detected at P0, suggesting that the upregulation of *Arnt* controlled genes is achieved by an increase in the formation of the heterodimer Arnt:AhR and not Arnt:Hif1a [37]. There was no enrichment of binding sites detected in the genes upregulated at P0.

*ELK1* and TFs associated with AHRHIF are known to change their expression during regeneration. The expression of *ELK1* was reported to increase 30 min after wounding cochlear hair cells with a laser, marking an early signaling event that occurs after epithelial damage [22]. Another TF that was increased after cellular insult was the AHRHIF TF, *ARNT* whose expression increased 24 h after exposing cochlear hair cells to neomycin, only to decrease again by 48 h, together with *HIF1A* and *AHR*. These time points reflect a change of expression in the supporting cells [22]. The transient increase of *ARNT* during regeneration resembles its transient expression pattern during normal inner ear development, between E13 and E17 in mouse cochlear epithelial cells [38]. Interestingly, the three TFs (*ARNT*, *HIF1A*, and *AHR*) were also reported to respond to tissue damage caused by a different toxic compound (TCDD [39]). *E2F1* is an important pro-apoptotic TF [40], and under some mitochondrial stress, it engages apoptotic signals to cause deafness [41]. Regulation of transcription during the cell cycle is under the control of E2 factors (E2Fs), often in cooperation with nuclear factor Y (NF-Y) [42], another TF highlighted in this comparison. In utricle hair cell regeneration [23], *E2F1* is changing its expression in a pattern that is associated with cell cycle genes. As mentioned, this TF and its targets are upregulated in E16.5, an age when we see enrichment for cell cycle activity.

### Transcription factors affecting expression change between tissues

In contrast to the genes differentially expressed during the development, the set of genes upregulated in the cochlea was enriched for binding sites for the motifs: HIC1, E2F, ZNF219, ZF5, UF1H3BETA, MOVO-B, MAZ, VDR, MAZR, MTF-1, c-Myc:Max, AP-2, CAC-binding protein, ETF, E47, Lmo2 complex, RREB-1, LBP-1, CP2/LBP-1c/LSF, and Spz1. TFs associated with E2F, ZF5, and MAZ were significantly upregulated in the cochlea, while TFs associated with MOVO-B, VDR, and Lmo2 complex were upregulated in the vestibule. TFs associated with 11 of these 20 motifs (LBP-1, Lmo2 complex, E47, E2F, ZNF219, ZF5, VDR, MTF-1, c-Myc:Max, AP-2, CP2/LBP-1c/LSF) have been previously reported to change their expression during the regeneration of inner ear sensory epithelia [22, 23].

Focusing on genes that are altered both during development and regeneration, the enrichment of E2F noted indicates the presence of proliferation in the cochlea at the relevant period of development. Given the role of this TF family in inducing proliferation, their involvement in hair cell regeneration is not surprising and is currently the focus of active research [43]. In contrast, *ZF5* is known primarily as a repressor of transcription and specifically as a regulator of cell cycle progression (through *c-myc* [44]), and cognitive development (through *FMR1* [45]). Thus, the upregulation of expression in the cochlea, where its targets are also upregulated, was unexpected. This might indicate the existence of an additional activating role for *ZF5*, or that another TF is activating the transcription of these targets, and *ZF5* is upregulated as part of a negative feedback loop. In avian hair cell regeneration, the expression of *ZF5* in the cochlea increases late in the recovery from neomycin damage, suggesting a role in cochlear hair cell differentiation. Another TF with the same pattern of expression during hair cell regeneration is *LMO2*. Enrichments for *LMO2* binding sites were found in the list of upregulated genes from both the cochlea and the vestibule. While the results of the regeneration experiment support a function for *LMO2* in the cochlea, the expression of the TF in our experiment was higher in the vestibule. A possible explanation for this duality could be that LMO2 interacts with different partners in the two tissues, and thus, a different subset of genes is increased in each case. The Lmo2 complex typically contains a single GATA factor and a single TAL1/E47 heterodimer, but the GATA factor can be replaced by an additional TAL1/E47 heterodimer, resulting in a change in the genes regulated [46]. As *Gata2* and *Gata3* are upregulated in the vestibule and *Tal1* is upregulated in the cochlea (DE q-values = 7.32 × 10^−18^, 1.67 × 10^−175^, and 5.29 × 10^− 7^, respectively), the complexes formed in each tissue might differ in composition. *VDR* is a transcription factor regulated by vitamin D levels [47]. Hypo- and hypervitaminosis D can cause sensorineural hearing loss [48]. The downregulation of the gene in the cochlea, where its targets are upregulated, suggests a repressor role for this TF, which is supported by existing literature [49]. In utricle hair cell generation [23], *VDR*’s expression peaks in the 54–72 h window after the aminoglycoside damage. This pattern makes it a candidate for playing a role in the phenotypic conversion process from supporting cells to hair cells in the vestibule. According to our experiment, it might fulfill a similar role in the vestibular development.

In the set of genes upregulated in the vestibule, we could detect enrichment for binding sites for the 21 motifs: HNF4, SREBP-1, NF-1, PEA3, TEF-1, AP-2rep, NF-kappaB (p65), LBP-1, LUN-1, E2A, PU.1, MyoD, Nrf2, Lmo2 complex, COUPTF, ISRE, HEB, E47, SMAD, AML-1a, and c-Ets-1. TFs associated with five motifs (TEF-1, PU.1, Nrf2, Lmo2 complex, and ISRE) were significantly upregulated in the vestibule, while TFs associated with four other motifs (PEA3, COUPTF, most SMADs, and AML-1a) were upregulated in the cochlea. TFs associated with 15 of the 21 enriched motifs (HNF4, SREBP-1, PEA3, NF-kappaB p65, LBP-1, E2A, PU.1, MyoD, Nrf2, Lmo2 complex, COUPTF, ISRE, HEB, E47, SMAD, c-Ets-1) displayed a change in expression during the regeneration experiments [22, 23].

The upregulation of TFs associated with the motifs *Spi1* [PU.1] and *Nfe2l2* [Nrf2] in the vestibule supports their role as inducers of transcription. However, the decrease seen in the cochlear expression in late (48 h) recovery from neomycin [22] suggests that their repression is required for proper differentiation of supporting cells to cochlear cells. *SPI1* is known to be involved in hematopoietic development and induces proliferation of immune cells [50] and therefore might upregulate the immune functions that are enriched in the vestibule. Similarly, *NFE2L2* can upregulate functions related to stress response and specifically to antioxidant defense [51]. The expression pattern of *Nr2f1* and *Nr2f2* associated with the COUPTF motif is in agreement with their suggested role as repressors of transcription, as they are downregulated in the vestibule, although the motif as a whole is enriched in the genes upregulated in the vestibule. Following laser damage, the expression of *NR2F2* increases in the cochlea for 3 h and an increase in cochlear expression is also evident in late (48 h) recovery from neomycin [22]. *Nr2f2* is known to work as a repressor of myogenesis, inhibiting *MyoD* [52], another TF whose targets are upregulated in the vestibule. Our data suggest that their repressive effect might have a role in cochlea development.

SMADs are intracellular proteins that transduce extracellular signals from transforming growth factor beta (TGF-β) ligands to the nucleus, where they activate downstream gene transcription [53]. Although TGF-β signaling is thought to be active in the cochlea, our results show rather that the downstream targets of this pathway are enriched in the vestibule. In order to address this issue, we examined the expression levels of individual SMADs. Most receptor-regulated SMADs (R-SMADs) were upregulated in the cochlea (*Smad1*, *Smad2*, *Smad5*, *Smad9*), in agreement with the hypothesis of higher TGF-β activity in the cochlea. However, inhibitors of this signaling pathway (*Smad6* and *Smad7*) were also upregulated in the cochlea, and with relatively high FCs (1.9 and 1.6, respectively), and may be responsible for decreasing the transcription of the downstream genes in the cochlea compared to the vestibule. The story becomes more complex with the two intracellular pathways involving SMADs. The R-SMADS *Smad2* and *Smad3* mediate the response to TGF-β ligands, which participate in the regulation of inner ear development by retinoic acid [54]. *Smad2* was upregulated in the cochlea, while *Smad3* was upregulated in the vestibule. In the regeneration experiment [22], *SMAD2* expression in the vestibule increased in a late response to neomycin damage in the utricle, emphasizing the importance of TGF-β signaling for vestibular differentiation. In a different pathway, the R-SMADS *Smad1*, *Smad5*, and *Smad9* mediate the response to bone morphogenetic proteins (BPMs), which are involved in generation of inner ear sensory epithelia [55], as well as chondrogenesis [56]. All three were upregulated in the cochlea, with *Smad9* showing a very impressive FC of 3.4. *SMAD9* was also increased in response to late neomycin damage in the cochlea [22]. This, together with its high cochlear levels, implies that it plays a role in cochlear differentiation.

*SPI1* and *SMAD9* also change in expression during utricle hair cell regeneration [23]. The patterns of the expression are complex. Notably, their maximal deviations from the control are at time point 66 h, where *SPI1* is upregulated and *SMAD9* is downregulated. These changes are of opposite directions to those observed in cochlear regeneration in [22], agreeing with the tissue-specific roles of the two TFs.

### Transcription factors affecting expression ratio change with age

In the set of genes for which the cochlea to vestibule expression ratio increases with age $\left(\frac{Cochlea}{Vestibule}\uparrow \right)$, we could detect enrichment for the binding sites for the motifs HNF4, E47, and a group of nuclear receptors (LXR, PXR, CAR, COUP, RAR), AP-4, and SMAD. The expression ratio of *Nr2f1*, a COUP TF, increased significantly in the same direction as its targets, which might have a positive downstream effect on retinoic acid receptor (RAR) signaling [57]. Interestingly, TFs associated with all the motifs changed their expression during the regeneration of inner ear sensory epithelia [22].

Retinoid signaling is critical during inner ear embryonic development, as well as in the postnatal maintenance of its function [58]. Both vitamin A deficiency and intake of excess retinoic acid (RA) during pregnancy have been shown to cause malformations in ear development. In rodents, in utero exposure of fetuses to RA negatively affected the semicircular canals and the cochlea. Key components in retinoid signaling show spatiotemporal expression patterns, and the interactions that excess RA interferes with are dependent on the developmental stage. KEGG enrichment of our DE genes showed that metabolism of RA was higher in the vestibule and at P0. Taken together with the motif enrichment, we deduce that retinoid signaling is important to both cochlear and vestibular development, with its role in the cochlea becoming more prominent in the period between E16.5 and P0. In the hair cell regeneration experiment, the cochlear expression of the retinoid receptor *RARA* decreased 24 h after neomycin damage, but by 48 h, *NR2F1* expression increased [22]. This later increase might mimic the increase in retinoid signaling seen in normal development.

Interestingly, we could detect enrichment of binding sites for AML-1a, LEF1, LBP-1, HEB, and POU6F1 in the set of genes for which the vestibule to cochlea expression ratio increases with age $\left(\frac{Vestibule}{Cochlea}\uparrow \right)$. The expression ratio of *Runx1* [AML-1a] increased significantly in the same direction as its targets. TFs associated with LBP-1 and HEB also changed their expression during the regeneration of inner ear sensory epithelia.

### Comparison with transcription factors known to enhance mammalian hair cell regeneration

Previous studies that induced hair cell regeneration by coordinated manipulation of multiple factors, showed a better efficacy for the ectopic expression of *ETV4*, *TCF3*, *GATA3*, *MYCN*, or *ETS2* in combination with *ATOH1* over the overexpression of *ATOH1* alone [18, 19]. In retrospect, our method identifies some of the TFs that were mentioned earlier for their ability to induce differentiation. Specifically, it singles out *Etv4* [PEA3] and *Tcf3* [E47]; *Gata3* is a partner of the highlighted *Lmo2*; and *Mycn* and *Ets2* have similar targets as *c-myc* [59] and *Ets1* [60], respectively. Moreover, four out of five of the TF genes are DE between the cochlea and the vestibule (*Etv4*, *Gata3*, *Mycn*, and *Ets4*; *q* value ≤ 0.05), which makes them good candidates for experimental interventions, as explained above.

### Change in the proportion of hair cells in sensory epithelia

Because of the difficulty of dissecting out the sensory epithelia and separating the hair cells from the adjacent supporting cells, all tissue samples of this type contain varying amounts of both hair cells and supporting cells. This complicates conclusions as to whether differential expression can be attributed to differences in the expression profiles or to variability in the cell mixture composition. In order to address this issue, we produced expression signatures of hair cells and supporting cells from a previous experiment [21] and used them to compute the proportion of each type in each preparation. We also evaluated the heterogeneity of cell types assuming that the cochlear sample is contaminated by cells from the vestibule (or utricle) and vice versa.

A different subset of genes was used to create the signatures for E16.5 and P0. For each age, we ranked the genes in decreasing order of expression variance across the four reference samples (cochlear and vestibular GFP+ and GFP− samples). We then took the expression of the first *k* genes in the list, where *k* equals 453 for E16.5, and 193 for P0. The value of *k* was chosen so that it minimized the estimated percentage of contamination in our mixed data, i.e., the estimated percentage of cochlear cells in vestibular samples plus the estimated percentage of vestibular cells in cochlear samples. We predicted that this heuristic approach would improve the overall prediction accuracy, although it did not directly optimize the precision of estimation of the percentage hair cells, which was our main goal. We used DeconRNASeq to estimate the mixing proportions [61].

**Fig. 5:**
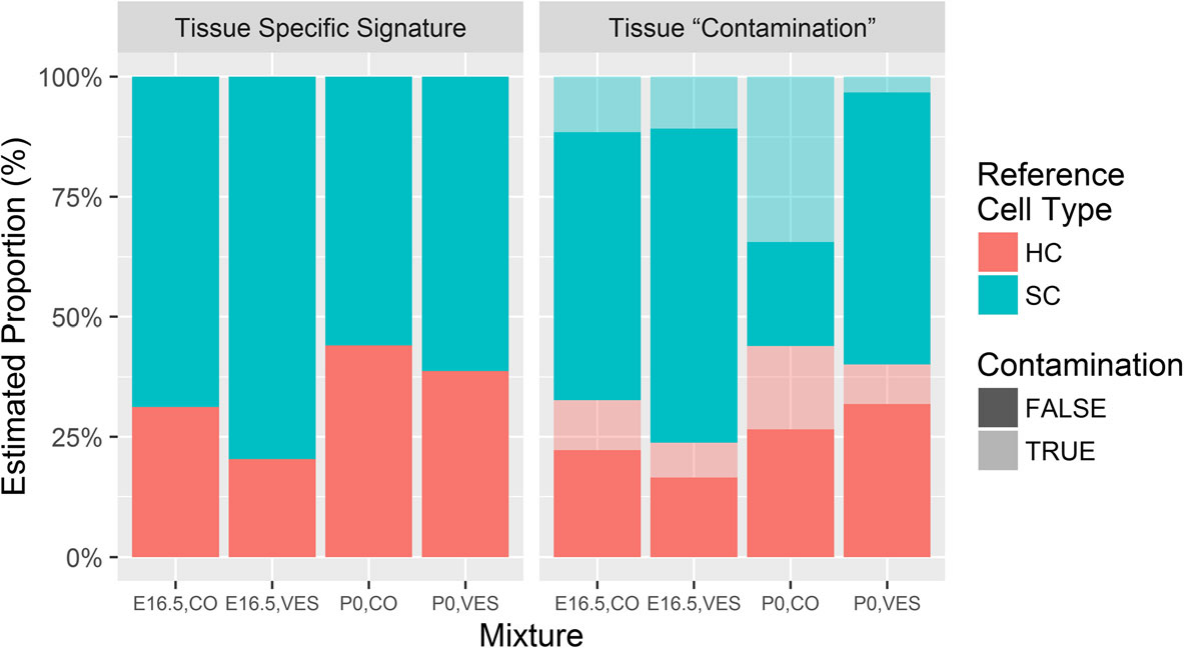
Estimated proportions of hair cells and supporting cells in various samples. This estimated proportion of each cell type in each of the groups is displayed in a stack bar chart, where the color of a stack identifies the cell type. On the left side, the cells composing a tissue were confined to cells originating from that tissue, without allowing cross-tissue contamination, whereas on the right side, cross-tissue contamination is assumed to occur. A light color indicates the amount of contamination. For example, focusing on the cochlear tissue at age P0 (P0.CO), the estimated proportion of hair cells, when contamination is not allowed, is 44.1% (in red). When contamination is allowed, the estimated proportion of hair cells decreases slightly to 44.0% and is composed of 26.6% cochlear hair cells (in dark red) and 17.4% contaminating vestibular hair cells (in light red). The three other samples show a majority of non-contaminated tissue (darker colors). HC hair cells, SC supporting cells

The estimated proportions of hair cells were similar in both scenarios (Fig. 5), which allows us to ignore the issue of possible tissue contamination. The estimated percentages (±SD) are 32.6 (± 1.6) and 23.8 (± 1.0) in the cochlea and the vestibule at E16.5 and 44.0 (± 1.1) and 40.1 (± 0.2) in the cochlea and the vestibule at P0, respectively. These results indicate that the percentage of hair cells is higher in the cochlea at both ages and increases with development in both tissues, with the increase in the vestibule being more prominent (1.9-fold increase compared to 1.4-fold in the cochlea). Strikingly, in all estimations, the percentage of supporting cells was higher than 50%, suggesting that these cells have a dominant influence on the expression profiles.

Even with the value of *k* selected to minimize the contamination, our calculation gives 51.8% contamination in the cochlea at P0. We are unsure how to interpret this high number. Possible causes for an overestimate of contamination could be (1) experimental noise, either in our data or the data used to generate the expression signatures at P0, or (2) inaccuracy of the deconvolution method when the signatures are similar. The similarity of the signatures of the same cell type in the cochlea and the vestibule can be seen by the high correlation values (*r* = 0.65, or 0.83 for signatures of hair cells and supporting cells, respectively).

## Discussion

In this study, we analyzed sensory epithelia RNA-seq data from mouse at E16.5 and P0, which correspond to developmental stages before and during the acquisition of mechanosensitivity. By exploring these data with considerations of developmental age and tissue type, we provided extensive information about the development of the inner ear. Moreover, we identified multiple transcription factors that are involved in transcriptional regulation, and a comparison with previous reports [22, 23] enabled us to focus on those that are also involved in regeneration of the avian inner ear after damage.

The sensory epithelium constitutes a heterogeneous tissue composed of hair cells and supporting cells, which cannot be easily separated by mechanical means. Although some previous experiments (e.g., [21]) employed FACS sorting to obtain pure populations, the analysis of data from the native tissue has the advantage of summarizing the expression of the hair cells and the milieu with which they interact. Although we did not physically separate the cells by type, we did estimate the contributions of each cell type by using expression deconvolution. Our results indicated a higher hair cell content in the cochlea compared to the vestibule, a content, which increased further during development in both tissues, albeit with a relatively larger increase in the vestibule.

Nearly 75% of the variation in the gene expression of our samples was explained by principal components associated with age (~ 47.5%) or tissue (~ 27.5%). Our analysis therefore focused on expression comparisons during development and across tissues. We also analyzed the more complex interaction of age and tissue. Our results showed that both cochlear and vestibular tissues become less proliferative with development and more differentiated in order to acquire the specialization required for their roles in sensory perception. According to our estimations, this specialization is accompanied by an increase in the relative proportion of hair cells in the sensory epithelia.

More surprising results were obtained from a comparison between tissues. While the cochlea was characterized mainly by neurological GO terms, the vestibule was shown to be enriched for vascular, structural, and immunological functions. Some of these differences could be attributed to alterations in the relative proportion of hair cells, which are presumed to be the dominant cell type in the cochlea. This finding has medical implications, as the higher vascularization of the vestibule, and its accessibility to immune cells, might impact the susceptibility of the tissues to ototoxic medications and inner ear infections.

With respect to the interaction of tissue and age, one notable finding was the delay in the development of sensory perception in the cochlea compared to the vestibule. This finding is supported by a delayed acquisition of mechanosensitivity in the cochlea (between P0 and P2 [62]) compared to the vestibule (between E16 and E17 [63]). In contrast, neuron projection and signaling at E16.5 is less developed in the vestibule than the cochlea, although the gap decreases by P0. This decrease can be attributed to the relatively larger increase in the proportion of hair cells in the vestibule compared to the cochlea.

Known DGs tended to be differentially expressed between the tissues. From E16.5 to P0, there was an increase both in expression and in the cochlear to vestibular expression ratio. In order to predict the probabilities of unidentified genes being yet undiscovered DGs, we built a classifier based on expression patterns. This classifier achieved a ROC score of 0.602 in predicting which genes are associated with deafness when validated by text mining tools. While the list of deafness-associated genes produced might not accurately reflect the genes that are essential for hearing, we believe it to be a good proxy for the true list. Ranking genes according to their algorithm estimated probability of being DGs can be useful in prioritizing candidate DGs in a real-world scenario, e.g., when multiple candidates arise from a familial segregation study.

We used enrichment analysis to identify TFs that are responsible for differences in expression between across tissues or developmental stages. Some of the TFs we identified as controlling expression were already known, e.g., the E2F family of TFs, which is responsible for promoting proliferation in the sensory epithelia and is controlled by retinoblastoma 1 during this stage of development [7], or the retinoic acid nuclear receptors, which are essential for the proper morphogenesis of the ear [58]. Our analysis not only strengthens the evidence connecting these known TFs to inner ear development, but also emphasizes their additional roles in hair cell regeneration in birds (see below). We also identified a number of TFs that do not have a known function in the inner ear. These include *Arnt*, which activates the transcription of its target genes in E16.5; COUP TFs, which we speculate to have a dual role, with *Nr2f2* in inhibiting myogenesis in the cochlea and *Nr2f1* in promoting retinoid signaling; and the hemopoiesis agent *Lmo2* [46], which we believe may interact with different coactivators in the vestibule and the cochlea.

To learn about the possibilities of regeneration of hearing in humans, it might be beneficial to mimic the regulation of transcription during a response to inner ear damage in birds and follow the ability of avian cochlear hair cells to regenerate [22]. Two experiments measured changes in TFs expression during such a response. The two complement each other, as the first [22] follows the response in both cochlear and vestibular tissues to either immediate laser damage or after a long exposure to ototoxic antibiotic, while the second [23] focuses only on vestibular response to an ototoxic antibiotic, but includes more frequent sampling, across a longer time period, using newer, more sensitive technologies, including RNA-seq. In these experiments, significant changes in hundreds of TFs were identified during the regenerative response. These factors were intersected with our list of developmental TFs, in order to highlight genes likely to be involved in either proliferation or differentiation. Although we could detect dozens of overlapping TFs, we focused only on those with differential expression, because this facilitates the interpretation of how the genes are regulated and suggests the possibility to influence this regulation through interventions.

To date, only a few TFs known to enhance mammalian cochlear hair cell regeneration were detected [18, 19]. For each of them, our method succeeded in detecting either the TF, a TF with a very similar motif or an interaction partner. Most of them showed differential expression, supporting our choice to further focus on TFs with this property. Moreover, our method highlighted the complex Arnt:AhR, which we believe is important in early development and undergoes a transient increase during regeneration of avian hair cells. We also concluded that an increase in the genes *Zbtb14* [ZF5], *Lmo2*, *Nr2f1*, *Nr2f2*, and *Smad9* and a decrease in *Spi1* [PU.1], *Nfe2l2* [Nrf2], and *Mafk* [Nrf2] are required for proper differentiation of the cochlea or hair cell regeneration. An increase in *Smad2* and *VDR* is involved in the parallel processes in the vestibule. Noteworthy, *Smad9* and *Spil* were DE in both avian regeneration experiments in directions that fit their suggested tissue specific roles.

The discussion has been concerned above with reprogramming of embryonic and neonatal tissue. Overcoming the limits of aging on reprogramming cochlear cells might be essential for clinical purposes because human cochleae become functionally mature neonatally and because sensorineural hearing loss is most prevalent in older adults [64]. Lately, it was shown that co-manipulation of *ATOH1* and *p27*^*kip1*^ creates new cochlear hair cells in adult mice [64]. In contrast to known functions of *p27*, the authors did not observe proliferation of supporting cells or converted hair cells following its deletion and concluded that *p27* plays a cell cycle-independent role in preventing *ATOH1*-mediated conversion of adult supporting cells to hair cells by repressing *GATA3* expression. Only a limited number of converted hair cells could be produced in the models suggested in the article, and some of the converted hair cells exhibited clear signs of apoptotic cell death. We believe that by inducing a more proliferative environment, the numbers of new hair cells generated would increase and their survival would improve. For this purpose, it might be beneficial to ectopically express TFs that are active in the highly proliferative age E16.5 and are also upregulated in hair cell regeneration, especially in the immediate response phase. According to these criteria, *Elk1* and *Arntl* are the most prominent candidates. Suppression of the pro-apoptotic *E2f1* might contribute as well.

## Conclusions

We found the cochlea to be more enriched in neurological functions, and to contain a higher percentage of hair cells than the vestibule, but also to display delayed development of sensory perception compared with the vestibule. The vestibule, on the other hand, was shown to be more vascular and more accessible to the immunological system. The majority of TFs we predict to be key regulators of the differentiation process have known functions that agree with this dichotomistic characterization. Selected TFs identified here may have potential as future candidates for inducing hair cell regeneration. Given the parallels between the mouse and human inner ear, in structure, function, genes, and mechanisms of pathogenesis leading to deafness, several of these candidates may be relevant for human hearing loss.

## Methods

### Generation of mRNA data

Data generation for E16.5 and P0 samples is described in our previous articles [24, 25, 65]. The datasets are available in the Gene Expression Omnibus (GEO) repository under accession numbers GSE97270 (E16.5) GSE76149 (P0) and are available on the gene Expression Analysis Resource web portal, gEAR, http://umgear.org/p?s=ace02363 (SVG); http://umgear.org/p?s=1e3f9408 (bar graph). Sequence data was analyzed as previously described [24].

### Principal component analysis

Principal components were calculated with R, after scaling and centering the log2-transformed RPKM values and plotted using ggbiplot (http://github.com/vqv/ggbiplot). Swamp (http://CRAN.R-project.org/package=swamp) was used to test the association between the principal components and annotations of sample age and tissue.

### Differential expression

Differential expression analysis was done using edgeR [66]. The design formula included the combination of age and tissue of each sample. The tested contrasts were the average difference between the two ages across tissues, the average difference between the two tissues across ages, and the difference of the differences at both ages. This last contrast is sometimes referred to as the interaction term of tissue and age. edgeR detection threshold was *q* value ≤ 0.05. An FDR correction was applied for each contrast separately.

### GO and KEGG enrichment analysis

We performed the enrichment analysis using the Expander software [67], exploring all GO ontologies, “biological process” (BP), “molecular function” (MF), and “cellular component” (CC) (corrected *p* value ≤ 0.05), and KEGG pathways (*q* value ≤ 0.01). For each contrast, we looked separately for enrichments in the set of genes upregulated and downregulated, using as a background set all the genes that were tested for differential expression.

### Illustrating age-tissue interacting GO terms

We calculated the expression ratios between the cochlea and the vestibule for E16.5 and P0 separately, using edgeR [66]. We then *z*-scored the ratios at each age, to allow a fair comparison of the ages. These ratios were used both to select which GO terms to display and to calculate a median ratio for each of these terms.

To select GO terms, we began with the lists of terms enriched in genes with increased cochlear to vestibular (C/V) or vestibular to cochlear (V/C) ratios between E16.5 and P0 (see GO and KEGG enrichment analysis). From each of these lists separately, we filtered only the GO terms for which the expression ratios of annotated genes are higher at P0 than at E16.5 (one-sided Wilcoxon signed rank test at the respected direction, *q* value ≤ 0.05). An FDR correction was applied for each list separately.

### TF enrichment analysis

We performed the enrichment analysis using PRIMA [68] with detection threshold *q* value ≤ 0.1. An FDR correction was applied for each list separately.

### Deafness genes expression patterns

One hundred forty DGs were manually curated from http://hereditaryhearingloss.org/ (updated 3/13/17). Using BioMart [69], we mapped 133 of the genes to mouse orthologs. Three genes were filtered out due to missing expression data of their homologs. To resolve multiple mapping, we preferably mapped to orthologs for which we have expression data. The DGs were annotated according to the type of deafness, as syndromic, non-syndromic, or mitochondrial. Genes that were associated with both syndromic and non-syndromic deafness were treated as if they were syndromic in subsequent analyses.

### Classifying deafness genes by expression

We built a classifier in order to categorize each gene as DG or non-DG. We are aware that some of the genes currently categorized as non-DGs are in fact DGs that have not yet been discovered. Our classifier thus learned to distinguish between positive and unlabeled genes. For every gene, the features for the classifier were (1) the averaged expression over all samples, in log counts per million (CPM), (2) the logarithm of the fold change (FC) of expression between the ages, (3) the logarithm of FC of expression between the tissues, and (4) the logarithm of the FC of the tissue expression ratio between the ages [i.e., log (cochlea to vestibule expression ratio at P0)/(cochlea to vestibule expression ratio at E16.5)]. This last feature represents the interaction of age and tissue. All four features were computed using edgeR [66]; specifically, the FCs were obtained from the model presented in the 'Differential expression' section. We trained the classifier with 75% of the genes, reserving the remaining 25% for testing purposes. Our classifier bagged over 1000 decision trees. Downsampling was used to account for the imbalance in the frequencies of the deafness and non-DGs (130 and 15,076 genes respectively). That is, to build each decision tree, we chose 130 non-DGs at random and used them together with all DGs in the building process. The R package caret was used for machine learning [70].

We used only 25 repeated training/test splits to compare the classifier with a classifier using the averaged RPKM values in each condition as features. Two thousand repeated splits were used to assign gene probabilities, although internal testing revealed that the ROC score reached a plateau after about 150 iterations. In each iteration, we used the classifier to predict the probabilities in the test set, corrected these probabilities for the undersampling bias, and corrected them again for the bias caused by the PU scenario. The correction methods are detailed below.

The correction of the biases did not affect the ranking of the genes in that iteration and was performed in order to produce well-calibrated probabilities. We averaged the probabilities over all iterations. The averaging caused minor differences in ranking between different methods of calibration, but the ROC score did not change significantly (*p* > 0.05, DeLong’s test for two correlated ROC curves [35]). We then assessed the calibration of the probabilities produced by each method. Under the assumption that most DGs are yet to be discovered, calibration curves that treat only known DGs as positive cases will falsely inflate the probabilities. For this reason, we downloaded lists of genes that were associated with hearing loss according to the text mining tools and assumed that these deafness-associated genes together with the known DGs comprise the full list of DGs. The annotation of DAGs and the comparison of the calibration are detailed below. An illustration of the classification process is provided in Additional file 1: Figure S6.

We then used these probabilities to build an improved classifier where *p*_*g*_ represents our estimation of the probability of gene *g*. We reran our bagging-like algorithm, but this time, we chose to treat a gene *g* as a positive example with probability *p*_*g*_ and as a negative example with probability 1 − *p*_*g*_. This reassignment was performed before each iteration, independently for each gene, and only for the unlabeled genes. Labeled genes were always treated as positive examples. This idea is inspired by [30] where the authors achieved slightly better results by rerunning their classifier with weights based on the initial probabilities learnt, after adjusting for the PU bias. Instead of reweighing the samples, we decided to reassign their classes, as reassignment (of only a few hundred genes) still allows us to perform undersampling. We again used 2000 repeated splits and averaged the probabilities over all iterations. We did not perform any bias correction until the end of the run, when we performed a correction only due to undersampling, as detailed below. We compared the ability of the initial classifier and the “rerun” classifier to predict DAGs among all unlabeled genes using DeLong’s test for two correlated ROC curves [35]. Assuming a considerable portion of DAGs are undiscovered DGs, we wished our algorithm to rank those higher than genes that are neither known DGs nor deafness-associated. It should be noted that the probabilities assigned by the classifiers to known DGs are ignored in this comparison, because the annotation of these genes as positive in the training of the initial classifier can lead to an artificial inflation of the probabilities assigned by the “rerun” classifier. An illustration of the classification process improvement is provided in Additional file 1: Figure S7.

Finally, we converted the mouse genes back to human genes and resolved multiple mapping by averaging the assigned probabilities.

### Calibration of the estimator

The calibration of the probabilities was tested using calibration plots produced with the R package caret. The prediction space was discretized into 11 bins. Cases with a predicted value between 0 and 0.09 fell in the first bin, between 0.09 and 0.18 in the second bin, etc. For each bin, the mean predicted value was plotted against the true fraction of positive cases, together with the 95% binomial confidence interval. If the model is well calibrated, the points should fall near the diagonal line. We also used the BS to measure probabilities calibration [71]. The lower the BS, the more accurate are the probabilistic predictions of a model. Let $\widehat{p}\left({y}_i|{x}_i\right)$ be the probability estimate of sample *x*_*i*_ to have class *y*_*i*_ ∈ {0, 1}. Then BS is defined as:

$$ BS=\frac{1}{N}\sum \limits_{i=1}^N{\left\{{y}_i-\widehat{p}\left({y}_i|{x}_i\right)\right\}}^2 $$

### Correcting undersampling bias

Undersampling creates an upward bias of the probabilities. To correct for this bias, we used the transformation suggested in [31] where *p*_*s*_ is the probability assigned by the model learnt on the balanced training set. *p’* is the bias-corrected probability obtained from *p*_*s*_:

$$ {p}^{\prime }=\frac{\beta {p}_s}{\beta {p}_s-{p}_s+1} $$

where *β* is the probability of selecting a negative instance with undersampling.

We used this method in two iterations. First, we adapted our PU classifier. Since we know whether each gene is positive or unlabeled (“negative”), then the estimation of *β* is trivial. We set $\beta =\frac{N^{+}}{N^{-}}$, with *N*^+^ = 130 and *N*^−^ = 15,076. Second, we adapted the “rerun” classifier, which used initial, well-calibrated probabilities as input. The expected number of DGs according to these input probabilities was *E*(*N*^+^) = 435. We thus set $\beta \cong \frac{E\left({N}^{+}\right)}{\mathrm{15,206}-E\left({N}^{+}\right)}$.

### Correcting positive-unlabeled bias

PU classifiers create a downward bias of the probabilities. If *x* is an example, then let *y* ∈ {0, 1} be a binary label. Let *s* = 1 if the example *x* is labeled and let *s* = 0 if *x* is unlabeled. According to [30], *p*(*y* = 1| *x*) = *p*(*s* = 1| *x*)/*c* where *c* = *p*(*s* = 1| *y* = 1). Our PU classifier estimates *p*(*s* = 1| *x*), the probability of the example to be labeled. In order to obtain an estimate for *p*(*y* = 1| *x*), the positivity probability, we need to divide the first probability by an estimate of *c*. Three estimators were suggested for *c*:

$$ {e}_1=\frac{1}{n}\sum \limits_{x\in P}g(x) $$

$$ {e}_2=\sum \limits_{x\in P}g(x)/\sum \limits_{x\in V}g(x) $$

$$ {e}_3=\underset{x\in V}{\max }g(x) $$

where *g*(*x*) = *p*(*s* = 1|*x*) is the posterior probability according to the PU classifier, *V* is the validation set, and *P* is the subset of examples in *V* that are labeled. We used the same set *V* for validation (estimating *c*) and for testing (estimating probabilities).

Methods e_1_ and e_2_ can give estimated probabilities higher than 1. For the calibration plots and calculation of BSs, we truncated them at 1 which gave us 1128 and 1897 probabilities that exceeded 1 for e_1_ and e_2_, respectively.

We note that *e*_1_ should theoretically have a lower variance than *e*_3_, since it involves averaging multiple samples instead of using just one [30]. However, we cannot assume that *e*_1_ is necessarily more accurate than *e*_3_, especially as the number of positive samples in a validation set used for *e*_1_ calculations is only 32 whereas the *e*_3_ set has a maximum of 3801 probabilities, and as such, might be more accurate. In practice, we used all three estimates and chose the one that produced the most calibrated probabilities to be *e*_3_.

### Deafness-associated gene annotation

We downloaded lists of genes that were associated with hearing loss according to the text mining tools DigSeE [32], DisGeNET [33], and DISEASES [34]. We searched the disease terms “Hearing Loss” in DigSeE and DisGeNET and the “Sensorineural Hearing Loss” in DISEASES. We then converted the human genes returned by the searches to mouse orthologs using BioMart [69]. In DisGeNET and DISEASES, an association has a score, but in DiGSeE, the association of gene *g* is characterized by the number of articles *n*_*g*, *a*_ and the number of sentences within articles *n*_*g*, *s*_ supporting it. We assigned this association the score ${n}_{g,a}+\frac{n_{g,s}}{\underset{x\in G}{\max }{n}_{x,s}+1}$, i.e., the number of sentences served as a tie breaker if two genes had the same number of articles. In order to calculate the ROC scores and BSs, we treated association as a binary trait and in order to demonstrate the effect of choosing different thresholds, we used Wilcoxon signed-rank test to compare the scores of genes with a probability above the threshold with the rest. The score of a gene not associated with deafness was set to 0. In this analysis, we also used a combined association score, which is the mean rank across the three lists of scores. We set the minimum “Combined” score to zero.

### Deconvolution of heterogeneous tissue samples

Using RNA-seq expression data from [21], we created an expression signature for each combination of tissue (cochlea/utricle), age (E16/P0), and type (GFP+/GFP−; GFP was specifically expressed in hair cells in the transgenic mouse used). For the process of deconvolution of heterogeneous tissue data, limiting signatures to a few hundred genes, which best separate the reference cell types, can provide accurate predictions [72, 73]. We therefore selected a separate subset of genes for each age using the following heuristics. First, we ranked the genes in decreasing order of expression variance across the four reference samples. Then, we took the first *k* genes in the list, with *k* selected to minimize a specific error in the deconvolution of our mixed data. The error measure used provides signatures that differentiate well between cochlear and vestibular origins of tissue (described below). Once *k* was determined, we built the expression signatures, and used them to assess the proportion of cells, under two different scenarios. In the first, we limited the cells composing a tissue to cells that originated from that tissue, while in the other, we allowed the inclusion of foreign cells mimicking a contaminated sample. The property we minimized in the selection of *k* was the estimated percentage of contamination in our mixed data under this second scenario, i.e., the estimated percentage of cochlear cells in vestibular samples plus the estimated percentage of vestibular cells in cochlear samples. We tested all possible *k*s in the range 1…1000. For E16.5, we chose the minimizing *k =* 453, but for P0, we ignored the first local minimum, which was narrow (~ 5 genes), and instead chose *k* = 193 (Additional file 1: Figure S8).

The expression data of the mixture was given in units of RPKM, and of the reference in counts per million (CPM). We did not normalize the reference data to the gene length, because the technique used in [61] of sequencing the 3′ end is not biased by gene length. Before building a signature, we filtered out genes for which the CPM was less than 1 in any of the conditions (within an age). The calculation of the variance in the expression of a gene was done on log-transformed expression.

We used DeconRNASeq to estimate the mixing proportions [61] with the default setting of the R package, except that we chose not to scale the data. We performed the deconvolution on the log-transformed expression. This is not generally recommended, specifically for microarray data, as it introduces a bias [74]. However, when we tried to work with the expression in the linear scale without log transformation, our results deviated extremely from what is known about the ratio of hair cell to supporting cells in both ages. To be specific, the estimated percentage of hair cells at E16.5 and P0 were ~ 12.5 and ~ 70% in both tissues. The gap is higher than expected, and, also, the second estimate is much higher than parallel quantities in other species. In adult humans between the ages of 27 and 67, 46.5% of the cells of the crista ampullaris are hair cells [75], and in hatched chicks, 28.2% of the cells in the utricular macula are hair cells [76]. Reference samples from E16 were used to estimate the proportions in our E16.5 samples. In addition, reference samples originating from the utricle were used to estimate the composition of our whole-vestibule samples. The estimation was done for each sample separately, and the predictions were subsequently averaged across each group.

## Additional files

Additional file 1: Supplementary Data. Supplementary table legends, figures, methods, and results. (PDF 2429 kb)

Additional file 2: Enrichment Analysis. Enrichments in genes differentially expressed between ages, tissues, and the interaction of age and tissue. (XLSX 234 kb)

Additional file 3: Deafness genes. Genes associated with deafness compiled from www.hereditaryhearingloss.org (updated 3/13/17), and the differential expression results for the mouse orthologs of the deafness genes. (XLSX 37 kb)

Additional file 4: Deafness genes prediction. Probabilities assigned to genes by the deafness genes prediction algorithm. (XLSX 4789 kb)

Additional file 5: Transcription factors affecting transcription. Motifs enriched in genes differentially expressed between ages, tissues, and the interaction of age and tissue, and the expression of the TFs associated with the enriched motifs. (XLSX 324 kb)

## Abbreviations

BS: Brier score
DAG: Deafness-associated gene
DE: Differentially expressed
DG: Deafness gene
E: Embryonic day
FC: Fold change
GO: Gene ontology
KEGG: Kyoto Encyclopedia of Genes and Genomes
P: Postnatal day
PCA: Principal component analysis
RA: Retinoic acid
TF: Transcription factor
