# Supplementary material for: Computational analysis of mRNA expression profiling in the inner ear reveals candidate transcription factors associated with proliferation, differentiation, and deafness
Source: Hum Genomics. 2018 Jun 22;12:30. doi: 10.1186/s40246-018-0161-7 (PMC6013912; doi:10.1186/s40246-018-0161-7)
Supplement: Supplementary file 1 — Supplementary Data. Supplementary table legends, figures, methods, and results. (PDF 2429 kb) [file 40246_2018_161_MOESM1_ESM.pdf]

Computational analysis of mRNA expression profiling in the inner ear reveals candidate transcription factors associated with proliferation, differentiation, and deafness

Kobi Perl, Ron Shamir and Karen B. Avraham

Supplementary Data

Contents

SUPPLEMENTARY TABLE LEGENDS....2
Table S1....2
Table S2....2
Table S3....2
Table S4....2
SUPPLEMENTARY FIGURES....3
Figure S1....3
Figure S2....4
Figure S3....5
Figure S4....6
Figure S5....7
Figure S6....8
Figure S7....9
Figure S8....10
SUPPLEMENTARY METHODS....11
Linear mixed models....11
Connecting motifs to target genes....11
Integration with avian sensory epithelia regeneration experiment....11
SUPPLEMENTARY RESULTS....13
Comparison of associated deafness genes lists....13
Known literature about enriched TFs in age comparison....13
Known literature about enriched TFs in tissue comparison....13
Known literature about enriched TFs in age-tissue interaction....16
REFERENCES....17

SUPPLEMENTARY TABLE LEGENDS

Table S1. Enrichment Analysis. Sheets: Age_(GO\KEGG), Tissue_(GO\KEGG), Tissue.Age_(GO\KEGG) – Enrichments in genes differentially expressed between ages, tissues, and the interaction of age and tissue, using either gene ontology (GO) or Kyoto Encyclopedia of Genes and Genomes (KEGG) enrichment analyses. Columns: Set – the set of genes checked for enrichments; Enriched with – enrichment term; #genes - number of genes annotated with the term; Raw p-value, Corrected p-value - p-value was corrected within a domain and a filter; Frequency in set (%) - frequency of genes annotated with the term in background; Gene List - the genes annotated for the enrichment term.

Table S2. Deafness Genes. Sheets: List – Genes associated with deafness compiled from www.hereditaryhearingloss.org (updated for 5/13/16); Expression – Differential expression for the mouse orthologs of the deafness genes. Common columns: HGNC – human symbol; 'List' columns: Gene (OMIM) – OMIM identifier; Group – deafness annotation, either Syndromic \ Nonsyndromic Genes \ Mitochondrial; Subgroup – more subtle subgrouping. 'Expression' columns: All.Groups, Chosen.Group – the type\s of deafness the gene is associated with, and the type selected for the analysis; All.Ensembls – associated human gene ids; All.Homologs, Chosen.Homolog – the mouse homologs of the gene, and the one selected for the analysis; logCPM – average expression across samples in logged-CPMs (counts per millions); Tissue.logFC, Tissue.FDR – the FC of expression between tissues (cochlea / vestibule), and the q-value of the differential expression; age.logFC, age.FDR - same for the comparison of ages (P0 / E16.5); tissue_age.logFC, tissue_age.FDR - same for the interaction of tissue and age (cochlea / vestibule ratio at P0 / same ratio at E16.5).

Table S3. Deafness genes prediction. Sheets: Mouse_Orthologs, Human_Genes – probabilities assigned to genes by the deafness genes prediction algorithm. The algorithm ran on mouse data but we provide also the associated human gene probabilities. Both sheets contain also scoring of the association between genes and hearing loss, according to different tools and methods (see Methods); Mouse_ROC, Human_ROC – ROC curve data for the prediction of deafness associated genes for both organisms. Only rows fitting local maxima of $Specificity + Sensitivity$ are stored. Mouse_Wilcox, Human_Wilcox – for increasing thresholds we store the p-value of comparing the association scores of genes above the threshold with those of genes below the threshold according to Wilcoxon rank sum test. Different scoring methods result in different p-values. Only rows fitting local minima of p-value are stored (according to the "Combined" scoring method).

Table S4. Transcription factors affecting transcription. Sheets: Age_Motif, Tissue_ Motif, Tissue.Age_ Motif – Motifs enriched in genes differentially expressed between ages, tissues, and the interaction of age and tissue; Age_TF_Expression, Tissue_TF_expression, Tissue.Age_TF_Expression – The expression of the TFs associated with the enriched motifs. Common columns: Set - the set of genes checked for enrichments; Enriched with – TRANSFAC motif name. Motif columns: #genes - number of genes with the motif in their promoter; p-value – significance of the enrichment; Enrichment factor – ratio of the fraction of genes with the motif in their promoter in the set to the analogous fraction in the background; Gene List – the genes with the motif in their promoter. TF expression columns: Symbol, Ensembl – associated TF; logFC - the FC of expression between the compared conditions; logCPM - average expression across samples in logged-CPMs (counts per millions); LR, PValue, FDR – likelihood ratio, p-value and q-value for the testing of differential expression of a TF between the compared conditions; DE – true if TF is differentially expressed between conditions (q-value≤0.05).

SUPPLEMENTARY FIGURES

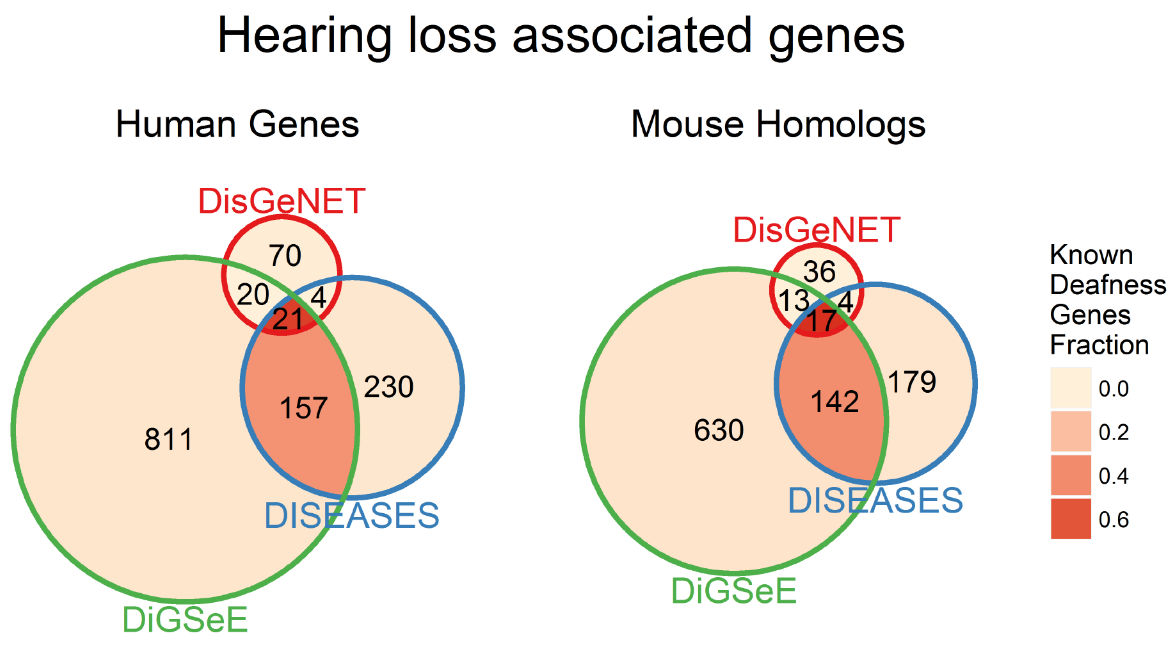

Figure S1. Number of genes associated with hearing loss. A Venn diagram of the number of human genes associated with hearing loss according to the text mining tools DigSeE, DisGeNET and DISEASES (see Methods). A darker region corresponds to a higher fraction of known deafness genes in the relevant set. Right: similar figure after converting the human genes to their mouse homologs.

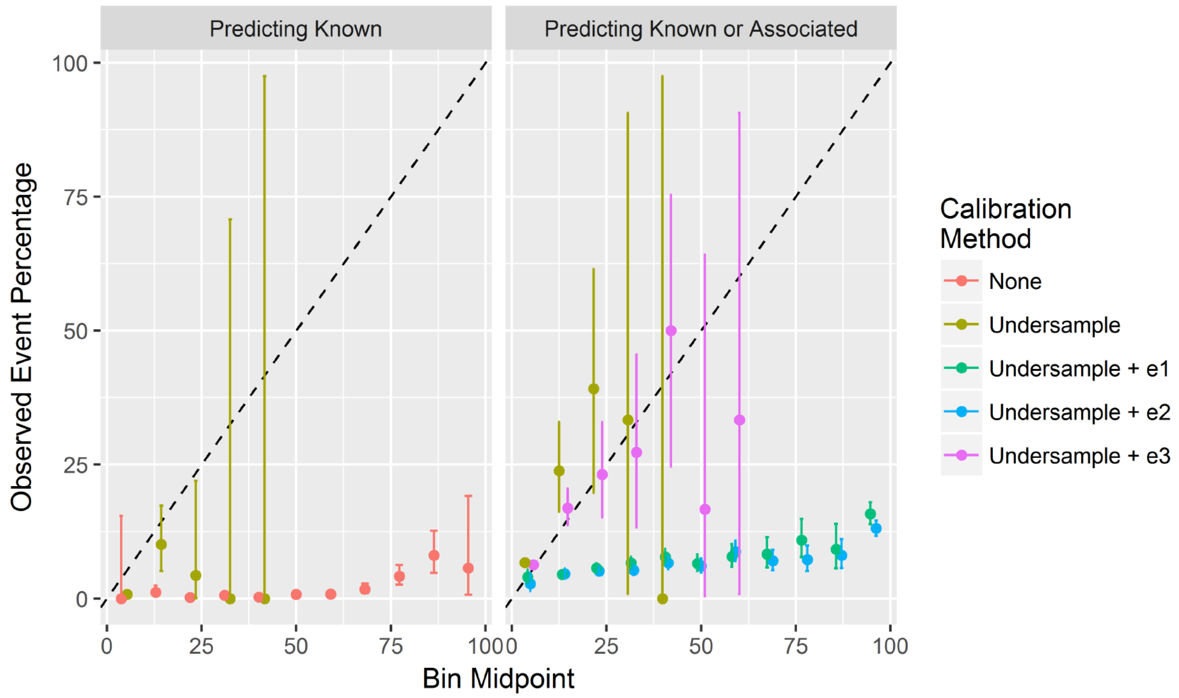

Figure S2. Probability calibration plots for classification models. The prediction space was discretized into 11 bins. Genes with predicted DG probability between 0 and 0.09 fell in the first bin, between 0.09 and 0.18 in the second bin, etc. For each bin, the mean predicted value was plotted against the true fraction of positive cases, along with the 95% binomial confidence interval. If the model is well calibrated the points should fall near the diagonal line. Left: The predicted probabilities of the PU classifier were either used directly (red) or calibrated for undersampling [1] (yellow). The plot shows how consistent the probabilities are with known deafness genes rates. Right: Three methods (e1, e2, e3) were used in order to calibrate the probabilities of the PU classifier for the classification of deafness genes versus non-deafness genes [2]. The calibration was performed after an initial calibration for undersampling. As the true label of an unlabeled gene is unknown, we use as proxy the association of such gene with deafness according to text mining tools. The calibration data for e1, e2, and e3 are colored green, blue, and purple, respectively. For some combinations of bins and calibration methods, there were no samples in the bin, and thus the mean predicted values were not plotted. For example, no gene was predicted to be a DG with a probability over 60% using the e3 method, so no purple points are plotted above this value in the x-axis.

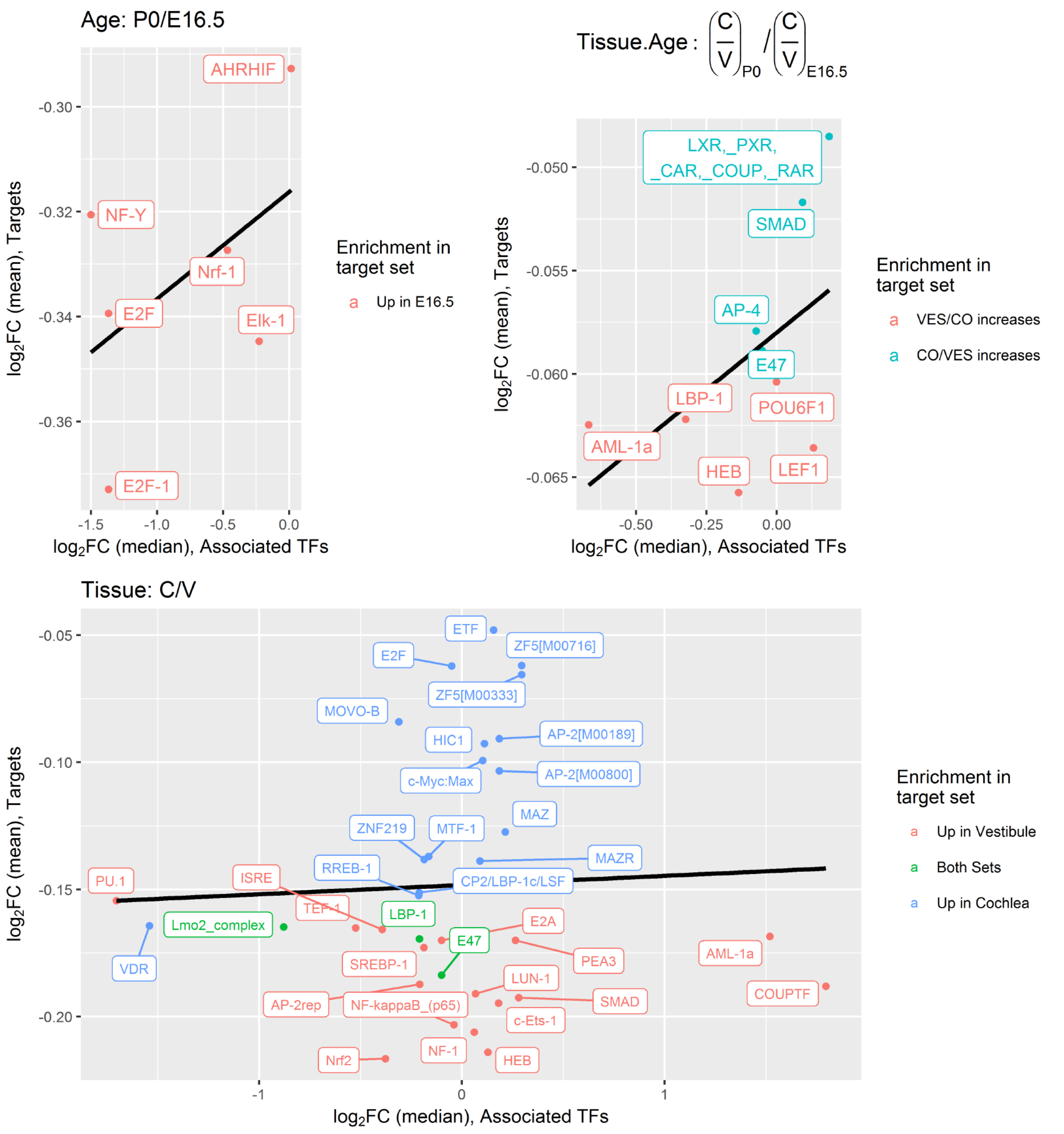

Figure S3. Expression of transcription factors and their targets. For the motifs enriched in the differentially expressed genes, we plotted the average log fold-change (FC) of the genes with the motif in their promoter, against the median logFC of the transcription factors associated with the motif. The subset of relevant motifs and the fold-changes were determined separately for each comparison: between ages (upper left), tissues (lower), and the interaction of age and tissue (upper right). The color indicates the gene set in which the motif was enriched. A linear regression line was added to each plot.

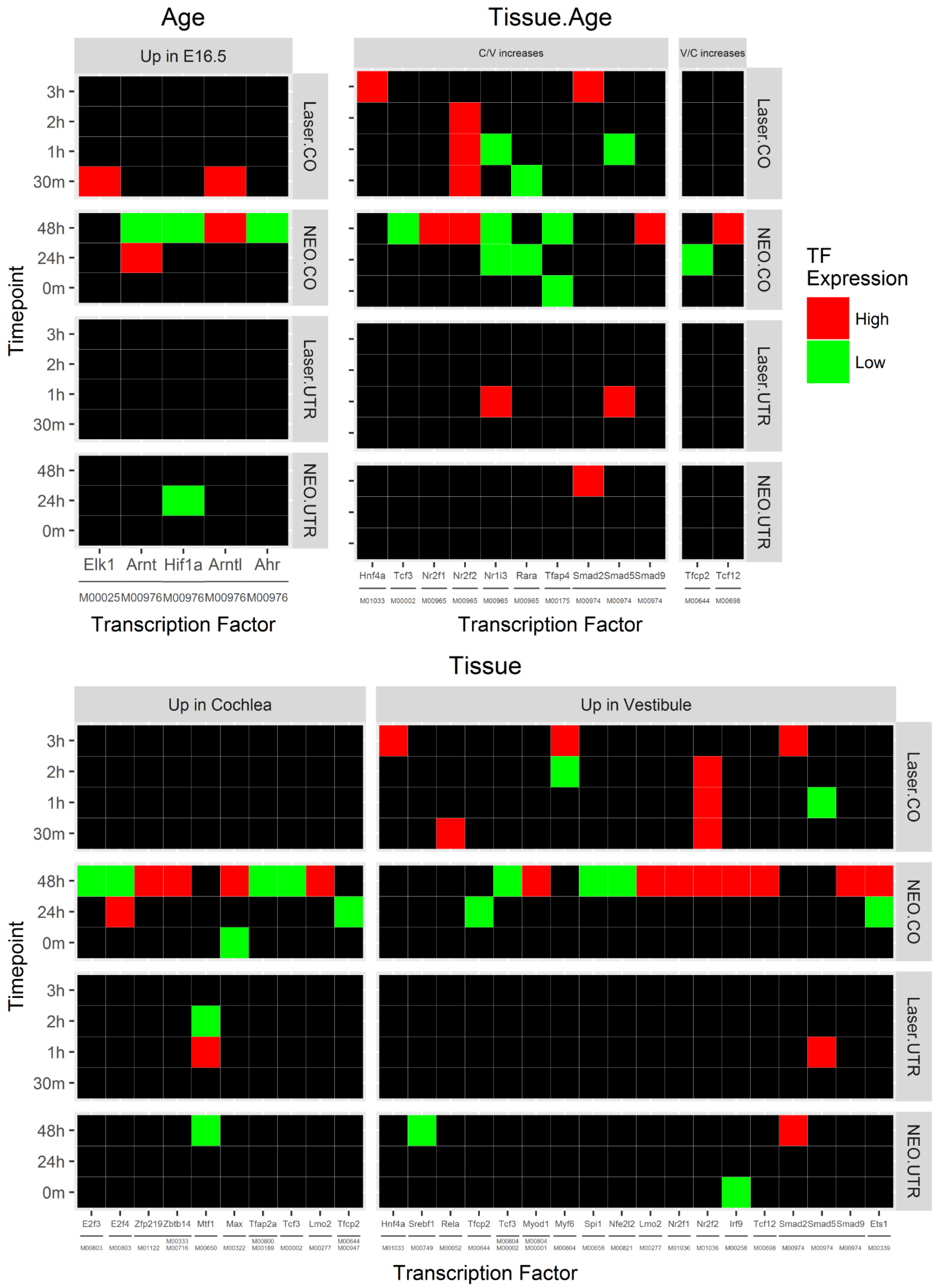

Figure S4. Transcription factors involved in avian hair cell regeneration according to Hawkins et al., 2007 [3]. For the motifs enriched in the differentially expressed genes, we illustrated how their associated transcription factors (TFs) changed during a previous avian sensory epithelia regeneration experiment. In that study, TF expression was measured in consecutive time points after the infliction of damage (either laser or Neomycin [NEO]) to an inner ear tissue (either cochlea [CO] or utricle [UTR]). Each tile represents a single measurement. The color indicates whether the TF expression was significantly high (red) or low (green) at this time point. The TFs and their associated TRANSFAC motifs [4] are indicated on the x-axis. The subset of relevant TFs was determined separately for each comparison: between ages (upper left), tissues (lower), and the interaction of age and tissue (upper right).

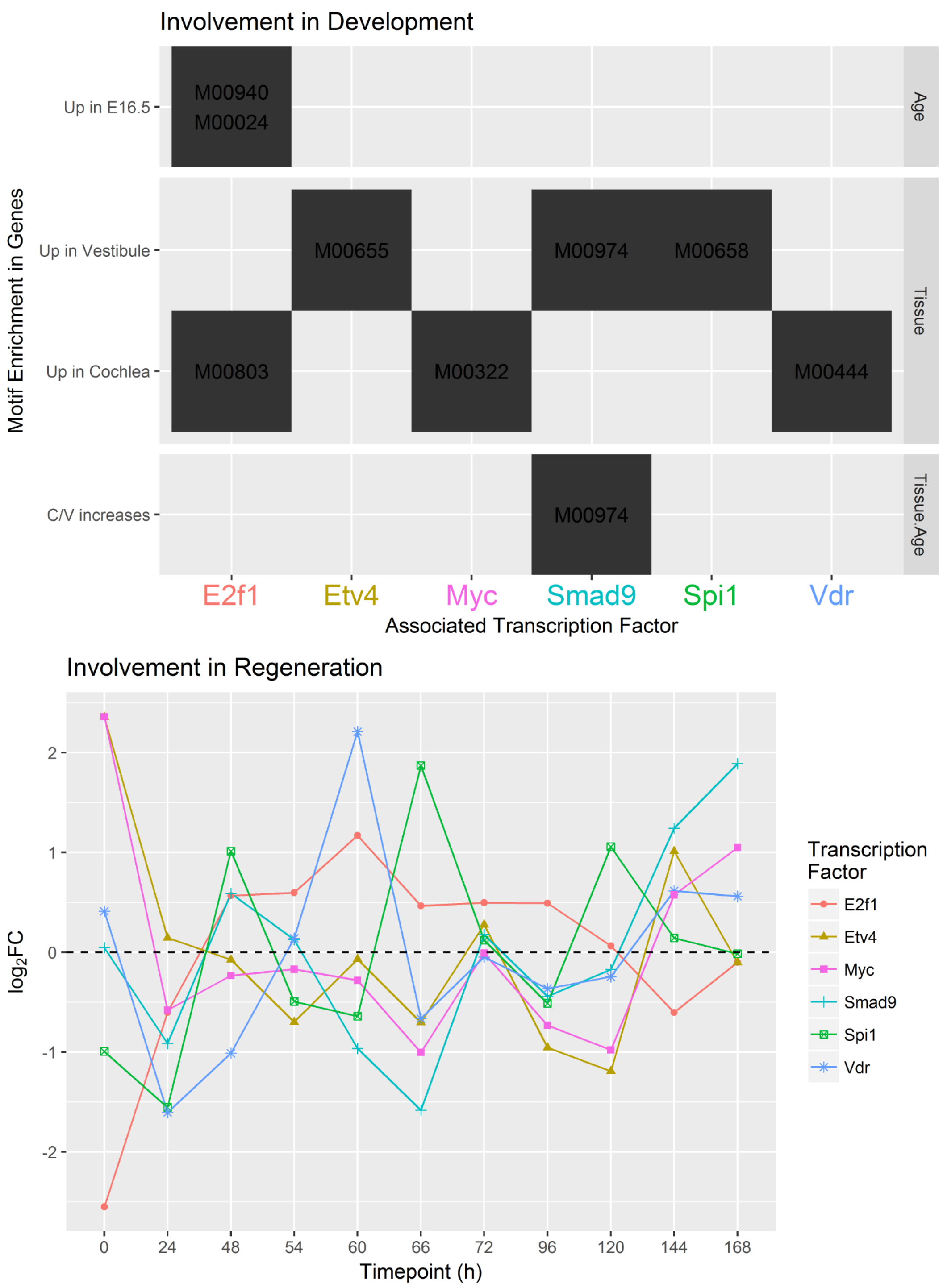

Figure S5. Concordance between transcription factors (TFs) regulating DE gene groups identified in our study and TFs involved in avian utricle hair cell regeneration according to Ku et al., 2014 [5]. In that study, RNA-seq was performed in consecutive time points after subjecting the whole utricles to aminoglycoside antibiotic treatment and extracting the sensory epithelia of the specimens (along with untreated control). A few hundred TFs involved in the process were identified, six of which overlapped with TFs regulating DE gene groups in our study. Bottom figure: the TFs' expression patterns, considered DE compared to control in the regeneration experiment. For each TF we plot the logarithm of the fold-changes (FCs; y-axis) between test and control specimens in the regeneration experiment for each timepoint (x-axis). Top figure: concordance between these six TFs and enrichment in DE genes in our experiment. For each TF (on the x-axis), we indicate in which lists of DE genes (on the y-axis), associated motifs were found (TRANSFAC [4] motif ids in dark tiles). The lists of DE genes were generated by comparison between ages (top), tissues (middle), and the interaction of age and tissue (bottom).

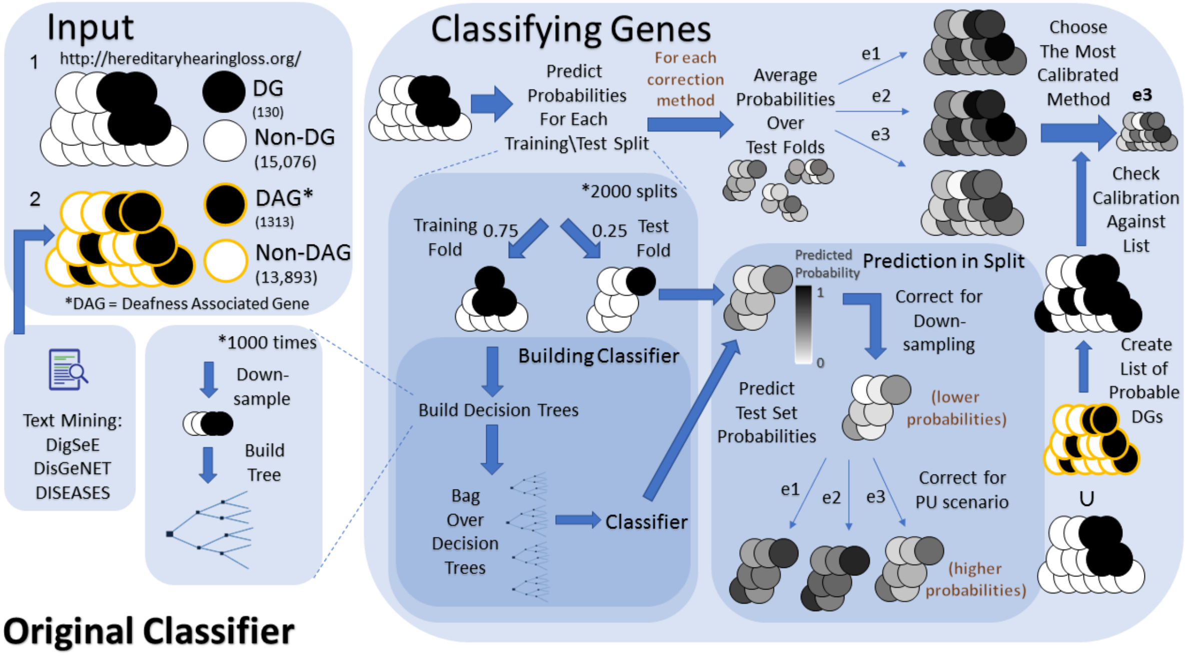

Figure S6. Illustration of the classification of genes as deafness genes. The input to this process is an assignment of genes to deafness genes and non-deafness genes, expression pattern data (not portrayed in the figure) and annotation of genes as deafness associated according to text mining tools. This last input type is only used in selecting the bias correction method. The output of the process is the predicted probability for each gene to be a DG.

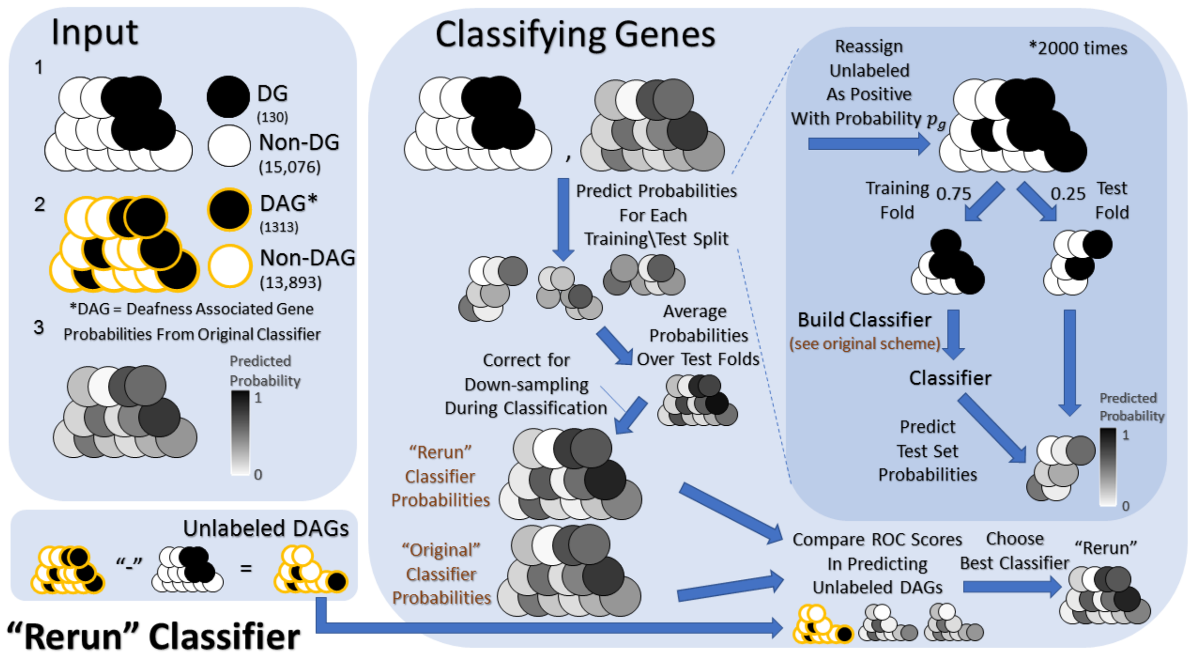

Figure S7. Illustration of the improved classification process of deafness genes. This "rerun" classifier uses the output probabilities of the original classifier (Figure S6) and refines them by randomly reassigning genes with unknown role in deafness as deafness genes during the learning process. The probability of a gene to be reassigned as a deafness gene is equal to the probability assigned to it by the original classifier.

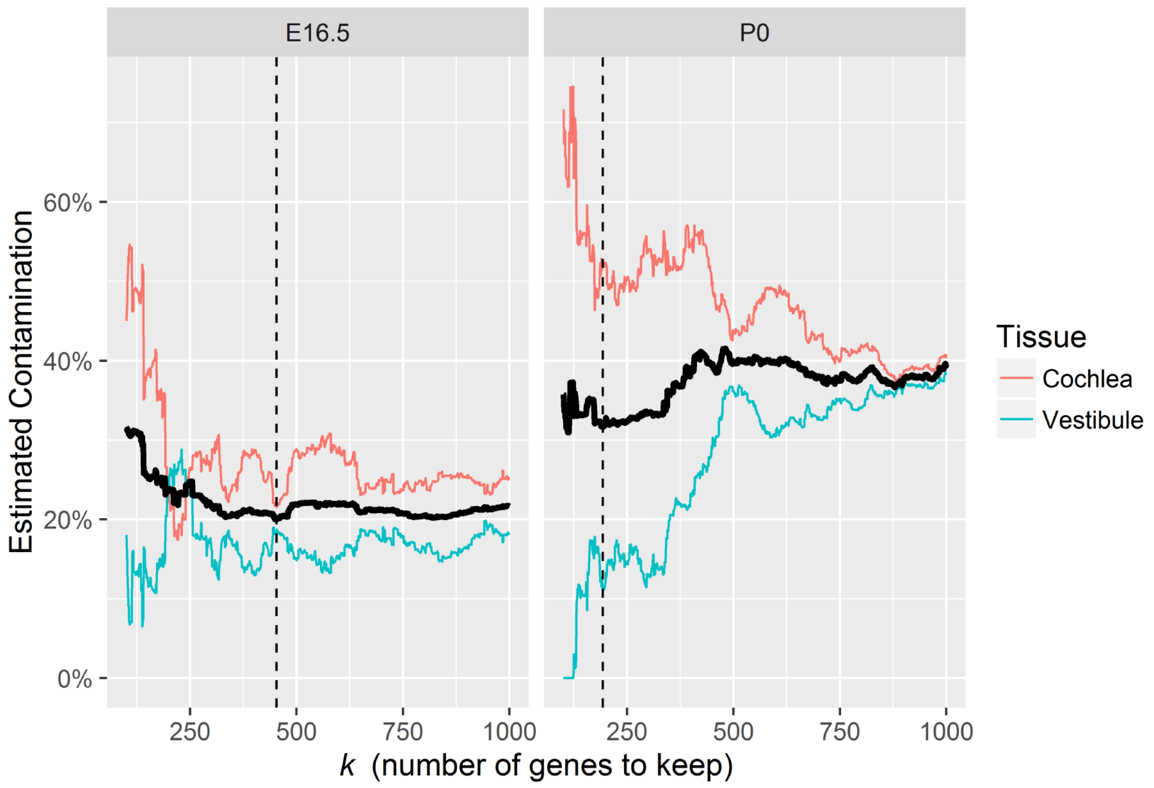

Figure S8. Choosing *k*: number of genes to keep for deconvolution. The estimated percentage of contamination is plotted against *k* for E16.5 and P0 (solid black line, left and right subfigures, respectively). The contaminations in cochlear and in vestibular samples are marked by the red and blue lines, respectively. The value of *k* chosen to minimize the contamination is marked by the dashed black line (see Methods for more details).

SUPPLEMENTARY METHODS

Linear mixed models

The following model was used to describe the expression level $E_{g,s}$ of gene *g* in sample *s*, which originated from tissue *t* at age *a*:

$$E_{g,s} = \alpha_g + \beta_s + T_{g,t} + A_{g,a} + I_{g,t,a}$$

The expression levels are in logged RPKM (reads per kilobase per million mapped reads). Random variables $T_{g,t}$ correspond to the effect of tissue identity on expression, $A_{g,a}$ correspond to the effect of age on expression, and $I_{g,t,a}$ correspond to a combined effect of tissue and age on expression. The parameters α_g correspond to the base expression levels of genes, and β_s correspond to the normalizing constants of expression between replicates.

Using lmer [6] we fitted this model to our data, and estimated the percentage of variance explained by each variance component. The high number of measurements did not allow fitting the model for all genes at once. Instead, we randomly selected 760 genes (5% of all genes) and fitted the model using their expression data. We performed this process 100 times. In 10 of those times, we used restricted likelihood ratio test to test whether the variance of the random effect $I_{g,t,a}$ is zero [7]. We reported the median *p-value* in the text.

Connecting motifs to target genes

We used the same mapping as PRIMA [8] uses by default. It contains for every gene and for every TRANSFAC motif [4] the number of hits (putative binding-sites) of the motif in the promoter of the gene (spanning from 1000 bp upstream the transcription start site [TSS] to 200 bp downstream the TSS). If there was at least one hit of a motif in the promoter of a gene, we considered the gene to be its target.

Integration with avian sensory epithelia regeneration experiment

In [3], transcription factor (TF) expression was measured in consecutive time points after the infliction of damage (either laser or Neomycin [NEO]) to an inner ear tissue (either cochlea [CO] or utricle [UTR]). We adopted the authors' thresholds for declaring a TF as DE in a single time point (FC≥1.2 and p-value≤0.05, compared with the background). Then, we found overlaps between TFs that are DE in at least a single time point in [3], and those arising from our enrichment analysis.

A similar process was performed for the newer regeneration experiment from the same group [5]. In this experiment the effect of an aminoglycoside antibiotic on gene expression in chick utricles was followed across a 7-day time course. Untreated utricles were cultured in parallel and served as controls. After treatment, the sensory epithelia of some specimens (along with untreated controls) were harvested for RNA-Seq in consecutive time points.

Once more, we adopted the authors' threshold for declaring a gene as DE (FC ≥1.8-fold and a p-value <0.05 at any one time point for treated versus control cultures), and then we looked for overlaps between TFs that are DE in the regeneration experiment, and those arising from our enrichment analysis. In [5], DE TFs were clustered by their expression dynamics into 16 centroids. For the overlapping TFs, we mention in the text the term used by the authors to describe the content of the assigned clusters (specifically, for *E2f1 and Vdr* the assigned clusters are described as 'cell cycle' and 'peaks at 54-72 hours', respectively; *Etv4 and Myc* were assigned to two different clusters of 'early effectors'; and *Smad9* and *Spi1* were assigned to two different clusters having a 'complex pattern').

SUPPLEMENTARY RESULTS

Comparison of associated deafness genes lists

The text mining tools used showed marked differences in the numbers of associated genes and percentages of known DGs within them (DiGSeE: 1009 genes, 10.5% are known DGs; DISEASES: 412 genes, 19.4% are known DGs; DisGeNet: 115 genes, 13.0%). A higher percentage of known DGs indicates a higher specificity. Thus, the largest list provided by DigSeE is relatively nonspecific, but might be more sensitive than the other lists, and the medium size list provided by DISEASES might not be as sensitive, but it is more specific. As expected, the higher the number of text mining tools associating a gene with deafness, the higher the probability of the gene to be a known DG (Figure S1).

Known literature about enriched TFs in age comparison

In the set of genes up-regulated at E16.5, we saw enrichments of binding sites for the motifs: Elk-1, Nrf-1, E2F-1, E2F, NF-Y, and AHRHIF. ***Elk1*** plays a role in regulation of neuronal cells, and possibly in synaptic plasticity and learning [9]. ***Nrf1*** is involved in regulating mitochondrial homeostasis [10], and over-expressing it reduces mitochondrial damage in an age-related hearing loss model [11]. For the **E2F** and **NF-Y** motifs, see main text. The motif **AHRHIF** is associated with the TFs aryl hydrocarbon receptor nuclear translocator (*Arnt*) and *Arnt2*, which are involved in regulation of developmental genes. In an earlier study [12], moderate to weak expression of *Arnt* was seen from E13 to E17 in the cochlear epithelial cells, mesenchyme, and also in the cartilage primordium of the petrous part of the temporal bone that surrounds the inner ear. The signal of *Arnt2* mRNA was seen both in the cochlear epithelial and mesenchymal cells from E13 onwards. See more about the AHRHIF family in the main text.

Known literature about enriched TFs in tissue comparison

In the set of genes up-regulated in the cochlea, we saw enrichments of binding sites for the motifs: HIC1, E2F, ZNF219, ZF5, UF1H3BETA, MOVO-B, MAZ, VDR, MAZR, MTF-1, c-Myc:Max, AP-2, CAC-binding protein, ETF, E47, Lmo2 complex, RREB-1, LBP-1, CP2/LBP-1c/LSF, and Spz1. The human gene ***HIC1*** is a tumor suppressor gene that acts as a transcriptional repressor [13]. Therefore, the enrichments of (the analog) *Hic1*'s targets in the cochlea up-regulated genes, suggest a higher activity of *Hic1* in the vestibule. During embryonic development *Hic1* is expressed mainly expressed in mesenchymal tissue, including precartilaginous condensations [14]. However, it has no known role in ear development. The deletion of *HIC1* is associated with the Miller-Deiker lissencephaly syndrome [14]. For the **E2F** motif, see main text. ***ZNF219*** is a translational repressor [15] with roles in regulating the neural protein alpha-synuclein [16] and in in chondrocyte

differentiation [17]. We can hypothesize that it is needed for the formation of cartilage in the inner ear, especially in the vestibule. ***ZF5*** is a putative c-myc repressor [18] associated with the fragile-X syndrome [19]. It has no known role in the inner ear. No data was found for **UF1H3BETA**. Yet, according to the TRANSFAC database (version used by PRIMA [8]), its consensus sequence contains the consensus sequence of another enriched motif (MAZ), suggesting a redundancy in enrichment results. **MOVO-B** is a splicing isoform of *Ovol2*, which is classified as a transcriptional repressor [20]; thus it is of no surprise that it is down-regulated in the cochlea, where its targets tend to overexpress. MOVO-B regulates testis-specific genes in the testis [21], and has roles in the development of the neural tube [22], the eye [23], the heart and the placenta [24], and possibly the skin [20]. In stem cells, it works downstream of the bone morphogenic pathway (BMP) to facilitate mesodermal differentiation and inhibit neural conversion [25]. It might fill a similar role in the vestibule (possibly in cooperation with R-SMADs *Smad1, Smad5, Smad9;* see main text). ***MAZ*** has a role in neuronal differentiation [26]. For the **VDR** motif, see main text. ***MAZR*** has a similar Zn finger motif as *MAZ* [27], and both activates the c-Myc promoter, however in [28] it is suggested that MAZR it acts as a transcriptional repressor. ***MTF-1*** is known for its role in heavy metal detoxification [29], and is essential for proper liver development but not essential for proper brain development. In epilepsy the level of zinc regulates the expression of specific calcium channels through this TF [30]; we note that calcium signaling is enriched in the cochlea. ***c-Myc:Max*** controls the balance of proliferation and differentiation in the ear as well as in other tissues; an intervention in its pathway was suggested for hearing restoration [31]. The **AP-2** family of TFs is expressed in primitive ectoderm and merging neural-crest cells [32]. Mutations within the human gene *TFAP2A* [AP-2] are associated with branchio-oculo-facial syndrome and inner ear malformation [33]. No data was found for **CAC-binding protein**. *TEAD2* [**ETF**] is important factor in regulation of neural development [34] and neuron proliferation [35]. It promotes cell proliferation and inhibits cell apoptosis [36]. The targets of *Tcf3* [**E47**] are enriched in both the cochlea and the vestibule upregulated genes. *Tcf3* is needed for hair cell development as a partner of ATHO1 [37], and also for neocortical development by repressing the Wnt signaling pathway [38], and to lymphocyte development [39]. It is reasonable to assume that this TF is involved in hair cell development in both sensory organs, but in the vestibule, in which the immunological activity is higher than the cochlea (see main text), *Tcf3* might also serve an immunological role. **Lmo2 complex** is also a motif enriched in both the cochlea and the vestibule analyses. *Lmo2* is essential for erythropoiesis [40], and also a possible role in the development of the cochlea [41], although its protein is undetectable by in-situ hybridization at E15.5 onwards. In the main text, we suggest *Lmo2* has a different partner in the cochlea and the vestibule. ***RREB1*** has a role in regulating cell adhesion and cell migration [42]. It has no known role in the inner ear. The

targets the **LBP-1** motifs are enriched in both the cochlea and the vestibule upregulated genes. LBP-1 proteins were primarily studied in the context of erythroid development [43]. ***Spz1*** known to specifically expressed in the testis [44], and was accordingly undetected in our experiment, suggesting this enrichment is a false positive.

In the set of genes up-regulated in the vestibule, we saw enrichments of binding sites for: HNF4, SREBP-1, NF-1, PEA3, TEF-1, AP-2rep, NF-kappaB (p65), LBP-1, LUN-1, E2A, PU.1, MyoD, Nrf2, Lmo2 complex, COUPTF, ISRE, HEB, E47, SMAD, AML-1a, and c-Ets-1 (see Main Text). ***HNF4*** is a TF regulating metabolism, cell junctions, differentiation and proliferation in liver and intestinal epithelial cells [45]. It is not expressed in the brain [46]. Its role in differentiation [47], as well as a possible role in inflammation [45], make it an interesting candidate in our system, although its levels were undetected in our experiment. ***SREBP-1*** regulates genes required for glucose metabolism and fatty acid and lipid production [48]; this last activity is dependent in retinoic acid [49], an important molecule in the inner ear [50]. ***NF-1*** is known to be essential for proper brain development in mouse [51, 52], but has no known role in the inner ear. **PEA3** is a downstream effector of FGF signaling, and has a role in ear development [53]. The enrichment of this motif in vestibular up-regulated genes cannot be easily explained, as the associated transcription factor *Etv4*, is up-regulated in the cochlea. The expression dynamics of this TF in utricle hair cell regeneration [5] shows a transient increase immediately after incubation with aminoglycoside antibiotic, which fits an early effector with a role in proliferation but not differentiation. ***TEF-1*** is essential for cardiac, skeletal, and smooth muscle development [54]; The term 'vascular process in circulatory system' appears in the enrichments of the genes up-regulated in the vestibule, and might indicate similarities in the development of the two systems. **AP-2rep** is responsible for repressing the AP-2 family of transcription factors [55]; In their turn they are responsible for cell-type-specific stimulation of proliferation and the suppression of terminal differentiation during embryonic development [32]. **NF-κB** contributes to the development and survival of the cells and tissues that carry out immune responses in mammals [56], and thus can be linked with the immune response enrichment in the vestibule. **LBP-1** is mentioned above, as it is enriched in the cochlea's up-regulated genes as well. ***LUN-1*** (a splicing variant of the *TOPORS* gene) expresses highly in the lung [57] and is associated with lung cancer [58], it also expresses highly in the CNS, although little is known on its role in neuronal tissue [59]. The ***E2A*** gene is encoding **E47** [60], which is mentioned above, as it is enriched in the cochlea's up-regulated genes as well. ***PU.1*** is a involved in hematopoietic development [61]; This once more can be related to the enrichment for immune response in the vestibule. ***MyoD*** is important for regulation of skeletal muscle gene transcription [62]; KO mouse for Myf5:MyoD show improper development of the vestibule; this is however attributed to a problem in transferring

mechanical stimuli needed for development and not a development problem within the inner ear [63]. **Lmo2** is mentioned above, as it is enriched in the cochlea's up-regulated genes as well. **COUP-TF**s are transcription factors, which have been shown to have functions in embryonic development. COUP-TFI is expressed mainly in the nervous system, and its targeted deletion leads to defects in the central and peripheral nervous systems, and COUP-TFII is highly expressed in the mesenchymal component of the developing organs [64]. See more about the COUP-TF in the main text. **ISRE** is stimulated by interferon, and affect cell proliferation, differentiation and regulation of the immune system [65]**;** This TF might be related to the enrichments we see for immune response in the vestibule. *TCF12* **[HEB]** plays part in embryonic myogenesis pathways [66], where it serves as a MyoD coactivator , and is also related to lymphocyte development [39], together with E47. For the **SMAD** family, see the main text. ***AML1-A*** is known to be essential for hemopoietic myeloid cell differentiation [67]; Hemopoiesis is upregulated in the vestibule. Also, it is known to interaction with SMADs and c-Ets-1 [68]. *Ets1* **[c-Ets-1]** promotes cellular differentiation in haemotopoietic cells, and in the vascular system, it promotes invasive behavior of endothelial cells and vascular smooth muscle cells [69].

Known literature about enriched TFs in age-tissue interaction

In the set of genes for which the cochlea to vestibule expression ratio is increasing with age $\left(\frac{Cochlea}{Vestibule}\uparrow\right)$, we see enrichments of binding sites for the motifs: HNF4, E47, a group of nuclear receptors (LXR, PXR, CAR, COUP, RAR), AP-4, and SMAD. ***HNF4*** was previously mentioned as a motif enriched in the genes up-regulated in the vestibule (see previous section). Its appearance in this list indicates some closure of a transcriptional gap between the cochlea and the vestibule. ***E47*** was also previously mentioned in the previous section. For **LXR, PXR, CAR, COUP, RAR**, see main text. *TFAP4* [**AP-4**] is involved in the regulation of cellular proliferation, stemness, and epithelial-mesenchymal transition [70]. Interestingly, it has a role in repressing neuron-specific genes in non-neuronal cells [71]. For the **SMAD** family, see the main text.

In the set of genes for which the vestibule to cochlea expression ratio is increasing with age $\left(\frac{Vestibule}{Cochlea}\uparrow\right)$, we saw enrichment of binding sites for: AML-1a, LEF1, LBP-1, HEB, and POU6F1. **AML-1a** and **HEB** were previously mentioned as motifs enriched in the genes up-regulated in the vestibule (see previous section). Their appearance in this list indicates some increase of a transcriptional gap between the cochlea and the vestibule. **LBP-1** was also previously mentioned in the previous section. **LEF1** is specifically expressed at early stages of B-cell differentiation and is essential for survival and proliferation [72].

REFERENCES

1. Dal Pozzolo A, Caelen O, Johnson RA, Bontempi G. Calibrating Probability with Undersampling for Unbalanced Classification. In: 2015 IEEE Symposium Series on Computational Intelligence. IEEE; 2015. p. 159–66.

2. Elkan C, Noto K. Learning classifiers from only positive and unlabeled data. In: Proceedings of the 14th ACM SIGKDD international conference on Knowledge discovery and data mining - KDD 08. New York, New York, USA: ACM Press; 2008. p. 213–20.

3. Hawkins RD, Bashiardes S, Powder KE, Sajan SA, Bhonagiri V, Alvarado DM, et al. Large Scale Gene Expression Profiles of Regenerating Inner Ear Sensory Epithelia. PLoS One. 2007;2:e525.

4. Matys V, Kel-Margoulis O V, Fricke E, Liebich I, Land S, Barre-Dirrie A, et al. TRANSFAC and its module TRANSCompel: transcriptional gene regulation in eukaryotes. Nucleic Acids Res. 2006;34 Database issue:D108-10.

5. Ku Y-C, Renaud NA, Veile RA, Helms C, Voelker CCJ, Warchol ME, et al. The transcriptome of utricle hair cell regeneration in the avian inner ear. J Neurosci. 2014;34:3523–35.

6. Bates D, Mächler M, Bolker B, Walker S. Fitting Linear Mixed-Effects Models Using lme4. J Stat Softw. 2015;67:1–48.

7. Scheipl F, Greven S, Küchenhoff H. Size and power of tests for a zero random effect variance or polynomial regression in additive and linear mixed models. Comput Stat Data Anal. 2008;52:3283–99.

8. Elkon R, Linhart C, Sharan R, Shamir R, Shiloh Y. Genome-wide in silico identification of transcriptional regulators controlling the cell cycle in human cells. Genome Res. 2003;13:773–80.

9. Besnard A, Galan-Rodriguez B, Vanhoutte P, Caboche J. Elk-1 a Transcription Factor with Multiple Facets in the Brain. Front Neurosci. 2011;5:35.

10. Evans MJ, Scarpulla RC. NRF-1: a trans-activator of nuclear-encoded respiratory genes in animal cells. Genes Dev. 1990;4:1023–34.

11. Fujimoto C, Yamasoba T. Oxidative stresses and mitochondrial dysfunction in age-related hearing loss. Oxid Med Cell Longev. 2014;2014.

12. Aitola MH, Pelto-Huikko MT. Expression of Arnt and Arnt2 mRNA in developing murine

tissues. J Histochem Cytochem. 2003;51:41–54.

13. Pinte S, Stankovic-Valentin N, Deltour S, Rood BR, Guérardel C, Leprince D. The tumor suppressor gene HIC1 (hypermethylated in cancer 1) is a sequence-specific transcriptional repressor: definition of its consensus binding sequence and analysis of its DNA binding and repressive properties. J Biol Chem. 2004;279:38313–24.

14. Grimm C, Spörle R, Schmid TE, Adler ID, Adamski J, Schughart K, et al. Isolation and embryonic expression of the novel mouse gene Hic1, the homologue of HIC1, a candidate gene for the Miller-Dieker syndrome. Hum Mol Genet. 1999;8:697–710.

15. Sakai T, Hino K, Wada S, Maeda H. Identification of the DNA binding specificity of the human ZNF219 protein and its function as a transcriptional repressor. DNA Res. 2003;10:155–65.

16. Clough RL, Dermentzaki G, Stefanis L. Functional dissection of the α-synuclein promoter: transcriptional regulation by ZSCAN21 and ZNF219. J Neurochem. 2009;110:1479–90.

17. Takigawa Y, Hata K, Muramatsu S, Amano K, Ono K, Wakabayashi M, et al. The transcription factor Znf219 regulates chondrocyte differentiation by assembling a transcription factory with Sox9. J Cell Sci. 2010;123 Pt 21:3780–8.

18. Sobek-Klocke I, Disqué-Kochem C, Ronsiek M, Klocke R, Jockusch H, Breuning A, et al. The human gene ZFP161 on 18p11.21-pter encodes a putative c-myc repressor and is homologous to murine Zfp161 (Chr 17) and Zfp161-rs1 (X Chr). Genomics. 1997;43:156–64.

19. Gulyy P V., Orlov S V, Dizhe EB, Kuteikin-Teplyakov KB, Ignatovich IA, Zhuk S V, et al. Roles of ZF5 and CGGBP-20 transcription factors in regulating expression of human FMR1 gene responsible for fragile X-syndrome. Cell tissue biol. 2010;4:54–62.

20. Lee B, Villarreal-Ponce A, Fallahi M, Ovadia J, Sun P, Yu Q-C, et al. Transcriptional mechanisms link epithelial plasticity to adhesion and differentiation of epidermal progenitor cells. Dev Cell. 2014;29:47–58.

21. Unezaki S, Nishizawa M, Okuda-Ashitaka E, Masu Y, Mukai M, Kobayashi S, et al. Characterization of the isoforms of MOVO zinc finger protein, a mouse homologue of Drosophila Ovo, as transcription factors. Gene. 2004;336:47–58.

22. Mackay DR, Hu M, Li B, Rhéaume C, Dai X. The mouse Ovol2 gene is required for cranial neural tube development. Dev Biol. 2006;291:38–52.

23. Lapan SW, Reddien PW. Transcriptome analysis of the planarian eye identifies ovo as a specific regulator of eye regeneration. Cell Rep. 2012;2:294–307.

24. Unezaki S, Horai R, Sudo K, Iwakura Y, Ito S. Ovol2/Movo, a homologue of Drosophila ovo, is required for angiogenesis, heart formation and placental development in mice. Genes to Cells. 2007;12:773–785.

25. Zhang T, Zhu Q, Xie Z, Chen Y, Qiao Y, Li L, et al. The Zinc Finger Transcription Factor Ovol2 Acts Downstream of the Bone Morphogenetic Protein Pathway to Regulate the Cell Fate Decision between Neuroectoderm and Mesendoderm. J Biol Chem. 2013;288:6166–77.

26. Okamoto S, Sherman K, Bai G, Lipton SA. Effect of the ubiquitous transcription factors, SP1 and MAZ, on NMDA receptor subunit type 1 (NR1) expression during neuronal differentiation. Mol Brain Res. 2002;107:89–96.

27. Kobayashi A, Yamagiwa H, Hoshino H, Muto A, Sato K, Morita M, et al. A combinatorial code for gene expression generated by transcription factor Bach2 and MAZR (MAZ-related factor) through the BTB/POZ domain. Mol Cell Biol. 2000;20:1733–46.

28. Fedele M, Benvenuto G, Pero R, Majello B, Battista S, Lembo F, et al. A novel member of the BTB/POZ family, PATZ, associates with the RNF4 RING finger protein and acts as a transcriptional repressor. J Biol Chem. 2000;275:7894–901.

29. Wang Y, Wimmer U, Lichtlen P, Inderbitzin D, Stieger B, Meier PJ, et al. Metal-responsive transcription factor-1 (MTF-1) is essential for embryonic liver development and heavy metal detoxification in the adult liver. FASEB J. 2004;18:1071–9.

30. van Loo KMJ, Schaub C, Pitsch J, Kulbida R, Opitz T, Ekstein D, et al. Zinc regulates a key transcriptional pathway for epileptogenesis via metal-regulatory transcription factor 1. Nat Commun. 2015;6:8688.

31. Kopecky B, Fritzsch B. The myc road to hearing restoration. Cells. 2012;1:667–98.

32. Eckert D, Buhl S, Weber S, Jäger R, Schorle H. The AP-2 family of transcription factors. Genome Biol. 2005;6:246.

33. Tekin M, Sırmacı A, Yüksel-Konuk B, Fitoz S, Sennaroğlu L. A complex TFAP2A allele is associated with branchio-oculo-facial syndrome and inner ear malformation in a deaf child. Am J Med Genet Part A. 2009;149A:427–30.

34. Kaneko KJ, Kohn MJ, Liu C, DePamphilis ML. Transcription factor TEAD2 is involved in

neural tube closure. Genesis. 2007;45:577–87.

35. Jacquemin P, Hwang JJ, Martial JA, Dollé P, Davidson I. A novel family of developmentally regulated mammalian transcription factors containing the TEA/ATTS DNA binding domain. J Biol Chem. 1996;271:21775–85.

36. Sawada A, Kiyonari H, Ukita K, Nishioka N, Imuta Y, Sasaki H. Redundant Roles of Tead1 and Tead2 in Notochord Development and the Regulation of Cell Proliferation and Survival. Mol Cell Biol. 2008;28:3177–89.

37. Su Y-X, Hou C-C, Yang W-X. Control of hair cell development by molecular pathways involving Atoh1, Hes1 and Hes5. Gene. 2015;558:6–24.

38. Kuwahara A, Sakai H, Xu Y, Itoh Y, Hirabayashi Y, Gotoh Y. Tcf3 represses Wnt-β-catenin signaling and maintains neural stem cell population during neocortical development. PLoS One. 2014;9:e94408.

39. Quong MW, Romanow WJ, Murre C. E protein function in lymphocyte development. Annu Rev Immunol. 2002;20:301–22.

40. Osada H, Grutz GG, Axelson H, Forster A, Rabbitts TH. LIM-only protein Lmo2 forms a protein complex with erythroid transcription factor GATA-1. Leukemia. 1997;11 Suppl 3:307–12.

41. Deng M, Pan L, Xie X, Gan L. Differential expression of LIM domain-only (LMO) genes in the developing mouse inner ear. Gene Expr Patterns. 2006;6:857–63.

42. Melani M, Simpson KJ, Brugge JS, Montell D. Regulation of cell adhesion and collective cell migration by hindsight and its human homolog RREB1. Curr Biol. 2008;18:532–7.

43. Katsura A, Kimura K, Hosoi K, Tomokuni Y, Nesori M, Goryo K, et al. Transactivation activity of LBP-1 proteins and their dimerization in living cells. Genes to Cells. 2009;14:1183–96.

44. Hsu S-H, Shyu H-W, Hsieh-Li H-M, Li H. Spz1, a novel bHLH-Zip protein, is specifically expressed in testis. Mech Dev. 2001;100:177–87.

45. Babeu J-P, Boudreau F. Hepatocyte nuclear factor 4-alpha involvement in liver and intestinal inflammatory networks. World J Gastroenterol. 2014;20:22–30.

46. Sladek FM, Zhong WM, Lai E, Darnell JE. Liver-enriched transcription factor HNF-4 is a novel member of the steroid hormone receptor superfamily. Genes Dev. 1990;4:2353–65.

47. DeLaForest A, Nagaoka M, Si-Tayeb K, Noto FK, Konopka G, Battle MA, et al. HNF4A is essential for specification of hepatic progenitors from human pluripotent stem cells. Development. 2011;138:4143–53.

48. Ferré P, Foufelle F. Hepatic steatosis: a role for de novo lipogenesis and the transcription factor SREBP-1c. Diabetes Obes Metab. 2010;12 Suppl 2:83–92.

49. Roder K, Zhang L, Schweizer M. SREBP-1c mediates the retinoid-dependent increase in fatty acid synthase promoter activity in HepG2. FEBS Lett. 2007;581:2715–20.

50. Romand R, Dollé P, Hashino E. Retinoid signaling in inner ear development. J Neurobiol. 2006;66:687–704.

51. Wong YW, Schulze C, Streichert T, Gronostajski RM, Schachner M, Tilling T. Gene expression analysis of nuclear factor I-A deficient mice indicates delayed brain maturation. Genome Biol. 2007;8:R72.

52. Driller K, Pagenstecher A, Uhl M, Omran H, Berlis A, Gründer A, et al. Nuclear factor I X deficiency causes brain malformation and severe skeletal defects. Mol Cell Biol. 2007;27:3855–67.

53. Barembaum M, Bronner-Fraser M. Pax2 and Pea3 synergize to activate a novel regulatory enhancer for spalt4 in the developing ear. Dev Biol. 2010;340:222–31.

54. Anbanandam A, Albarado DC, Nguyen CT, Halder G, Gao X, Veeraraghavan S. Insights into transcription enhancer factor 1 (TEF-1) activity from the solution structure of the TEA domain. Proc Natl Acad Sci U S A. 2006;103:17225–30.

55. Imhof A, Schuierer M, Werner O, Moser M, Roth C, Bauer R, et al. Transcriptional regulation of the AP-2alpha promoter by BTEB-1 and AP-2rep, a novel wt-1/egr-related zinc finger repressor. Mol Cell Biol. 1999;19:194–204.

56. Hayden MS, West AP, Ghosh S. NF-κB and the immune response. Oncogene. 2006;25:6758–80.

57. Chu D, Kakazu N, Gorrin-Rivas MJ, Lu HP, Kawata M, Abe T, et al. Cloning and characterization of LUN, a novel ring finger protein that is highly expressed in lung and specifically binds to a palindromic sequence. J Biol Chem. 2001;276:14004–13.

58. Oyanagi H, Takenaka K, Ishikawa S, Kawano Y, Adachi Y, Ueda K, et al. Expression of LUN gene that encodes a novel RING finger protein is correlated with development and progression of non-small cell lung cancer. Lung Cancer. 2004;46:21–8.

59. Jeffery IB, Madden SF, McGettigan PA, Perrière G, Culhane AC, Higgins DG. Integrating transcription factor binding site information with gene expression datasets. Bioinformatics. 2007;23:298–305.

60. Frasca D, Nguyen D, Riley RL, Blomberg BB. Decreased E12 and/or E47 Transcription Factor Activity in the Bone Marrow As Well As in the Spleen of Aged Mice. J Immunol. 2003;170:719–26.

61. Celada A, Borràs FE, Soler C, Lloberas J, Klemsz M, van Beveren C, et al. The transcription factor PU.1 is involved in macrophage proliferation. J Exp Med. 1996;184:61–9.

62. Tapscott SJ. The circuitry of a master switch: Myod and the regulation of skeletal muscle gene transcription. Development. 2005;132:2685–95.

63. Rot I, Kablar B. The influence of acoustic and static stimuli on development of inner ear sensory epithelia. Int J Dev Neurosci. 2010;28:309–15.

64. Pipaón C, Tsai SY, Tsai M-J. COUP-TF Upregulates NGFI-A Gene Expression through an Sp1 Binding Site. Mol Cell Biol. 1999;19:2734–45.

65. Gongora C. A unique ISRE, in the TATA-less human Isg20 promoter, confers IRF-1-mediated responsiveness to both interferon type I and type II. Nucleic Acids Res. 2000;28:2333–41.

66. Zhao P, Hoffman EP. Embryonic myogenesis pathways in muscle regeneration. Dev Dyn. 2004;229:380–92.

67. Tanaka T, Tanaka K, Ogawa S, Kurokawa M, Mitani K, Yazaki Y, et al. An acute myeloid leukemia gene, AML1, regulates transcriptional activation and hemopoietic myeloid cell differentiation antagonistically by two alternative spliced forms. Leukemia. 1997;11 Suppl 3:299–302.

68. Massagué J, Wotton D. Transcriptional control by the TGF-beta/Smad signaling system. EMBO J. 2000;19:1745–54.

69. Dittmer J. The biology of the Ets1 proto-oncogene. Mol Cancer. 2003;2:29.

70. D'Annibale S, Kim J, Magliozzi R, Low TY, Mohammed S, Heck AJR, et al. Proteasome-dependent degradation of transcription factor activating enhancer-binding protein 4 (TFAP4) controls mitotic division. J Biol Chem. 2014;289:7730–7.

71. Kim M-Y, Jeong BC, Lee JH, Kee HJ, Kook H, Kim NS, et al. A repressor complex, AP4

transcription factor and geminin, negatively regulates expression of target genes in nonneuronal cells. Proc Natl Acad Sci U S A. 2006;103:13074–9.

72. Erdfelder F, Hertweck M, Filipovich A, Uhrmacher S, Kreuzer K-A. High lymphoid enhancer-binding factor-1 expression is associated with disease progression and poor prognosis in chronic lymphocytic leukemia. Hematol Rep. 2010;2:e3.
